# Global and Local Ancestry and its Importance: A Review

**DOI:** 10.2174/0113892029298909240426094055

**Published:** 2024-05-09

**Authors:** Rangasai Chandra Goli, Kiyevi G. Chishi, Indrajit Ganguly, Sanjeev Singh, S.P. Dixit, Pallavi Rathi, Vikas Diwakar, Chandana Sree C, Omkar Maharudra Limbalkar, Nidhi Sukhija, K.K Kanaka

**Affiliations:** 1 ICAR-National Dairy Research Institute, Karnal, 132001, Haryana, India;; 2 ICAR-National Bureau of Animal Genetic Resources, Karnal, 132001, Haryana, India;; 3 ICAR- Indian Institute of Agricultural Biotechnology, Ranchi, 834010, Jharkhand, India;; 4 Central Tasar Research and Training Institute, Ranchi, 835303, Jharkhand, India

**Keywords:** Admixture, AIMs, ARG, local ancestry, post admixture selection signatures

## Abstract

The fastest way to significantly change the composition of a population is through admixture, an evolutionary mechanism. In animal breeding history, genetic admixture has provided both short-term and long-term advantages by utilizing the phenomenon of complementarity and heterosis in several traits and genetic diversity, respectively. The traditional method of admixture analysis by pedigree records has now been replaced greatly by genome-wide marker data that enables more precise estimations. Among these markers, SNPs have been the popular choice since they are cost-effective, not so laborious, and automation of genotyping is easy. Certain markers can suggest the possibility of a population's origin from a sample of DNA where the source individual is unknown or unwilling to disclose their lineage, which are called Ancestry-Informative Markers (AIMs). Revealing admixture level at the locus-specific level is termed as local ancestry and can be exploited to identify signs of recent selective response and can account for genetic drift. Considering the importance of genetic admixture and local ancestry, in this mini-review, both concepts are illustrated, encompassing basics, their estimation/identification methods, tools/software used and their applications.

## 1 INTRODUCTION

One of the foremost critical experiences from the period of cutting-edge genomics is the ubiquity of migration and admixture throughout animal history. As individuals migrate on a moderate to large scale, this allows the interchange of genes between at least two previously isolated groups. As a result, admixed populations are produced, giving rise to populations with ancestors from different origins, and the continuous portions of the genome inherited from a population are known as admixture segments or migrant tracts. Admixture segments are invisible, and the only way to determine their existence is through genomic information; this process is known as admixture deconvolution or ancestry painting [1]. Admixture is the quickest evolutionary mechanism to significantly alter the makeup of a population, and the admixed groups are still understudied in population genetics despite their prevalence and significance [2]. In order to illustrate the extremes of the process by which admixed groups are generated, two admixture dynamics models have been given. First is the Hybrid Isolation (HI) model, and second is the Continuous Gene Flow (CGF) model [3, 4]. According to the HI model, admixing happens instantly in just one generation without further input from either parental population; as a result, Admixture Linkage Disequilibrium (ALD) order of 10 to 20 cM is formed in a single generation and gradually degrades over time through independent assortment and locus recombination. The CGF model infers that the admixture happens at a stable pace in each generation from the contributions of one or all parental populations to the admixed population, and ALD rises with each generation. However, once the admixture fraction reaches 0.5, further mixing will actually cause the ALD to fall since more gene flow will turn the admixed population into the introgressing parental population. Over the history of animal breeding, admixing of different breeds has short-term benefits *i.e*., exploiting the notions of complementarity and heterosis of various traits resulting in the increased frequency of heterozygotes, which can hide harmful mutations or allow expression of overdominance, thereby lessening the detrimental impacts of genetic bottlenecks [5]. Also, an introduction of novel genotypes that are not present in parental populations could be crucial in some situations; these new genotypic combinations can yield transgression phenotypes that are far outside of parental norms. Long-term benefits of admixture influence contemporary genetic and phenotypic diversity, which may cause differences in adaptability to the environment and the development of diseases among the populations [6]. In fact, the increased genetic diversity brought about by admixture serves as the basis for local adaptation in recently settled ecosystems. Generally, admixture levels and ancestry are estimated using pedigree records; however, with the addition of genomic data, these estimations can be done with greater accuracy than with pedigree information alone [7]. Genetic admixture analysis in a population enables geneticists to divide individuals in a community into discrete groups based on specific genome-wide markers that are subsequently linked to biological entities. These admixing rates can be explored at several levels, ranging from the individual to the population, *i.e*., at the population, individual, or specific chromosomal areas (locus level). By utilizing genomic methods, the genetic mix of distinct breeds and whether they are purebred, graded, or crossbred can be determined [8]. It is also feasible to establish the historical and geographical origins of these breeds by recognizing their recent or distant mixing [9]. Genomic investigations can also be used to determine their divergence and mixing. In humans, genetic admixture has been widely explored during the preceding decades [10, 11]. However, admixture investigations of other species have only recently begun [12].

## 2 USE OF MARKERS IN ADMIXTURE STUDIES

Mostly in populations maintained at different locations, the breed composition and population structure are estimated on the basis of pedigree records, assuming strictly the halving nature of inheritance across various progeny generations or more remotely on the basis of farmer’s assessments and assumptions. The four grandparents of an individual are considered to have contributed a quarter each of the genomes of an animal, great grand-parents contribute one-eighth each, and so on, according to this method of calculation. However, across various farms and mainly in developing countries, human errors in recording, calculation and applying of various statistical procedures project major hindrances to the accurate pedigree and parentage/ancestry estimation. In most of the instances, the pedigree records remain unreliable and/or unavailable as well. The Mendelian and other genetic principles along with the genetic recombination phenomenon also pose a major threat to accurate interpretations of parentage and population structures. The process of recombination occurring across generations results in chunks of genomes being present as a mosaic in the genome, emanating from various ancestral populations [13]. The F1 generation, resultant of the admixture of two lineages, initially contains huge chunks of chromosomes evolving from the parental population, maintaining their individual identity at the genomic level. However, with further inter-breeding, the intact chromosomal chunks get mixed by the process of recombination and become mosaics in terms of chromosomal segments from input populations. It is the undisturbed chunks of chromosomes that, if exploited, are sure to provide a definite idea of admixture and the constituent of populations on a comparable basis.

The pedigree-based analysis is unable to depict the true inbreeding levels in each animal as the true allele frequencies in the base population remain unknown. Variation or genetic diversity is termed the lifeline of genetic studies and raw material of evolution. It provides the base material on which geneticists can work, and the same is needed for adaptation and speciation of livestock populations across the globe. Genetic structure, diversity and individual admixture analysis have helped in improving breeding tools for livestock improvement in developed countries. At the molecular level, there are several sources of variation and genetic diversity that are prevalent within a breeding group, ranging from recombination and mutation to others. The meticulous, analytical studies on closely linked markers will provide insights into how the parental haplotypes present themselves after several generations of crossbreeding. With routine and galloping progress in next generation sequencing and allied techniques, a huge help is promised to researchers as genome-wide assays have become a practice now. This allows for cost-effective and genome-wide characterization of various species for genetic diversity and population structure studies. A huge amount of data can now be generated for the characterization of population genetic diversity in various livestock species. Genomic markers have already been used to assess the depth of genetic variation among various livestock breeds across multiple species [14, 15]. These genetic markers notably include microsatellite and SNP variants.

## 3 MICROSATELLITE

Microsatellites are direct tandem repeating DNA sequences that range from one to six base pairs(bp) long. Hence, microsatellite markers are additionally known as simple sequence repeats (SSR). The genetic basis for these repeats could be faults in DNA replication or unequal crossing over during meiosis, and information from microsatellites is often related to repeat length [16-18]. Microsatellite-based genotyping is the method of choice for genetic characterization because of its high mutation rate, high polymorphism, codominance, and uniform dispersal at the genomic level, which aid in (i) the explanation of the total scale of genetic diversity within species. and (ii) the development of objective standards for conservation and a genetic enhancement scheme [19].

## 4 SINGLE NUCLEOTIDE POLYMORPHISM

A single nucleotide polymorphism (SNP) is a minor genetic change or variant that can arise within the DNA sequence of an individual. These are binary markers (biallelic) with less variability than multiple allele loci but are the most abundant as they occur once every 300 nucleotides on average in different species. This characteristic has allowed us to forecast the correlations of SNP markers to several critical economic variables in different animals. Myostatin (MSTN) has been related to animal double muscling, Bovine Leukocyte Adhesion Deficiency (BLAD), Complicated Vertebral Malformation (CVM), and Congenital Muscular Dystonia (CMD) [20, 21]. During the past few decades, SNPs have been explored singly or in groups of 10-15 in substantial numbers to develop some characteristic connections with an important trait prevalent in different animals.

SNP markers are robust tools in population genetics to assess selection signatures for breed classification purposes and for understanding various other diversity measures due to their genomic abundance and accessibility and include more than 90% of all differences between individuals [22-24]. SNP testing can reveal genetic ancestry by comparing DNA with reference populations from around the world [24]. It can provide information about your ethnic background and the regions where your ancestors likely originated from [25]. SNPs are used as genetic markers in a number of applications, such as genomic selection, quantitative trait loci (QTL) mapping, and marker-assisted selection (MAS) [26-28]. They contribute to the creation of breeding methods for desirable traits by offering insights into genetic diversity both within and between populations [29]. SNPs play a crucial role in deciphering the genetic underpinnings of intricate characteristics and diseases, facilitating the identification of genes linked to resistance to disease and other important traits [30].

## 5 USAGE OF MICROSATELLITE MARKERS *VERSUS* SNPS IN POPULATION STUDIES

Traditionally, microsatellite markers have been employed to measure breed variety, differentiation, introgression, and level of admixing [31]. Throughout the last few decades, microsatellite markers have been used across livestock species, and reliable results have been produced in the same context. After the dominance of microsatellite markers in genome-wide studies, SNPs have now emerged as important third-generation markers and act as a substitute for microsatellites in studies on different aspects of population genetics [32]. With the advent of density-based SNP panels, it has become extremely easy to conduct genome-wide studies on livestock species. On the basis of SNP, one requires much smaller sample sizes to obtain comparatively accurate allele frequency estimates. One study by Shi *et al.* revealed that samples as small as four individuals were enough to provide reliable genome-wide data based on SNP panels [33]. Another study by Frkonja *et al.* reported similar results with profound accuracy even with subsets of 10 animals for predictions of admixed individuals (correlations of 0.997–0.999) [34]. On the other hand, the same studies based on microsatellite markers require considerably larger sample sizes than SNPs to get better results. Among various species, the number of studies regarding the population structure and admixture analysis using microsatellite markers has been significantly high [24]. According to FAO, a restricted set of microsatellite loci, typically thirty (30) in number, should be targeted [35]. McKay *et al.* stressed the significance of abundant SNP markers in genetic diversity studies in order to accurately complement the standardized thirty (30) microsatellite markers [36]. This is reported as a consequence of the decreasing information content of specific SNP loci, however, due to the additional properties of SNP markers, they are being preferred nowadays. These properties include their robustness, cost-effectiveness, automatic allele calling, minimal mutations, prevalence across a genome and biallelic nature, that enable them to be detected by employing computerized methods [37]. These properties make them the markers of choice for genome-wide studies on different aspects of population genetics. SNPs are also thought to be the better candidates for the purpose of diversity studies; they are more abundant, genetically stable and easily responsive to complex analytical procedures when applicable [38]. Regarding the number of SNPs to be covered, about 500,000 SNPs may need to be established on genome-wide SNP maps for humans and up to 300,000 significant as well as effectively spaced SNP loci for cattle [39, 40]. De Roos *et al.* determined that 50,000 SNPs are required for studies on one breed, and about 300,000 SNP markers are required if the study is conducted across diverged breeds. Still, genome-wide studies on association mapping using a relatively lower number of SNP markers have produced successful results [41, 42]. This has eventually led us to a trend of SNP panels being designed and validated from 10K (10,000 SNP variant markers) to 777K (777,000 SNP variant markers) for several species. The rapid development of these panels aided in the acquisition of new information about the population structure and genetic diversity among the cattle population. On increasing the density from 50k to 777k, Gunia *et al.* reported that no significant effect was found on the accuracy of genome-based results [43]. The establishment of two large assemblies comprising a bovine genome would likely accelerate research on population structure and genetic diversity in cattle species [44]. The Bovine HapMap consortium has established itself as a launch pad for further studies on genetic diversity and population structure. The consortium data, based on a survey of 501 animals from 19 worldwide taurine (*Bos taurus*), indicine (*Bos indicus*), and crossbreds (taurine X indicine) populations, has just been released, and this assay covered approximately 30,000 SNP markers from the whole bovine genome [45].

## 6 ANCESTRY INFORMATIVE MARKERS

**Fig. (1):**
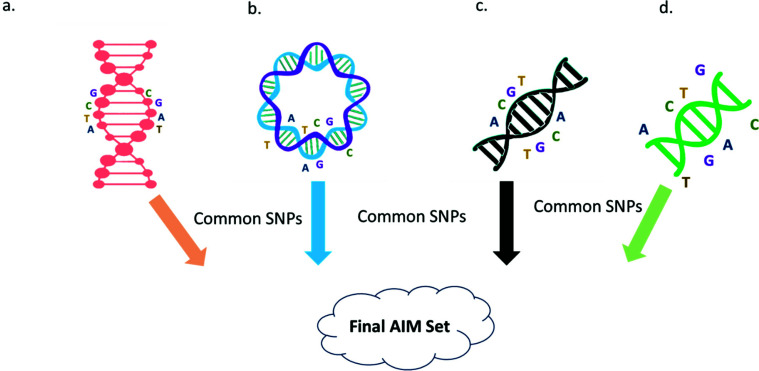
Inference of Ancestry informative markers (AIMs) panel. **a,b,c,d** are genotypes/marker set derived from different genotyping platforms and common SNPs are extracted to get AIMs. AIMs indicate population origin from DNA samples, aiding admixture research and individual biogeographical ancestry determination. Biallelic SNPs, prevalent for their abundance and ease of genotyping, are commonly used AIMs, requiring optimization in marker density to resolve ancestral transitions accurately.

The fraction of genetic material passed down from each pioneer group is referred to as ancestry. Ancestry Informative Markers (AIMs) are DNA markers that can indicate the probable origin of a population from a DNA sample if the original individual is unknown or unwilling to reveal their ancestry [46]. Any marker (STRs, SNPs) can be used, but biallelic SNPs are the most commonly used as they are numerous, regularly spread across the genome, and easily genotyped (Fig. **1**). AIMs are mostly utilized for admixture research and determining individual biogeographical ancestry (I-BGA) [47]. According to one study, while investigating admixture, a higher proportion of biallelic markers (SNPs) (4-10X) are necessary to obtain the same results with regard to effectiveness and precision as multi-allelic markers (microsatellites) and this issue can be solved by employing principal component approach by minimizing the dimension of variables [48]. Lewis *et al.* revealed that in most cases, the number of genetic markers necessary for ancestry interpretation may be reduced to 1.5% of the initial number of SNPs with an accuracy of 92% [49]. Admixture panels are made up of ancestry markers having significant information content that is evenly distributed across the genome, and the optimal density of the panel is dictated by the size of the ALD blocks, which are determined by the number of generations following the admixing event. As generations increase, ALD decays and linkage equilibrium is restored, resulting in smaller ALD blocks. Smaller ALD blocks necessitate higher marker intensity to differentiate chromosome ancestry transitions caused by meiotic crossover occurrences [50]. The most critical prerequisite for the admixture mapping panels is a group of genetic markers that offers information about the ancestry origin of each allele at each locus. Furthermore, markers must be distributed throughout the genome, autonomous, and sufficiently numerous to resolve ancestral transition from one ancestral chromosomal state to the next [50].

There are several approaches for determining the marker’s information content. There are two distinct and basically different methods. One method is to assess the mapping power of individual loci or a group of loci using available software [51, 52]. This software has its own set of benefits and drawbacks, but the most critical constraint is the computational constraint, although there are no restrictions on the number of loci or individuals to be studied [53]. The second approach is to rank loci solely based on their accuracy, *i.e*., the Marker’s information content, which refers to the amount of information a locus possesses about an individual's lineage. The introduction of informative markers minimizes the number of markers required for proper allocation [54]. Several measures/criteria of marker’s informativeness are proposed, such as Shannon information content, Delta, Pairwise Wright's F_ST_ by Wright, Global Wright's F_ST_ by Wright, Pairwise Weir and Cockerham F_ST_ by Weir and Cockerham, Global Pairwise Weir and Cockerham F_ST_ by Weir and Cockerham, and Informativeness for assignment (In) [54-58]. In recent years, a fresh data mining approach known as FIFS - Frequent Item Feature Selection was developed based on the identification of the most relevant markers from population genomic data using frequent items [59]. It is a modular approach that consists of two key components. The first identifies the most common and distinct genotypes in each community examined. The second one selects the best of them to provide useful SNP subsets.

## 7 APPROACHES FOR ADMIXTURE ESTIMATION THROUGH THE USE OF BIOINFORMATICS AND STATISTICAL TOOLS

There are two primary categories of methodologies used to determine the ancestry of an admixed population namely, global ancestry and local ancestry-based methods. The goal of global ancestry is to calculate the ancestral contributions made by each constituent population to crossbred populations. These estimations are based on an examination of marker variation distributed across the entire genome. The detection of global ancestry in any admixed population can be done using either a model-based approach or a non-parametric approach [60, 61].

## 8 MODEL-BASED APPROACH

The model-based technique finds chromosomal segments and chunks that are still intact throughout the ancestry after coming from the base population. The proportions of various breeds in the current population can be quantified with the aid of the identification of these chunks [62]. STRUCTURE and ADMIXTURE are the two most widely used bioinformatics tools for model-based global ancestry analysis [63, 64]. Both of these programs operate using a model that assumes that Hardy-Weinberg equilibrium and Linkage equilibrium exist across these loci and utilize ancestry portions and population allele frequencies derived from genotypic data [65, 66]. When admixture takes place, the contributing parental populations’ allele frequencies are combined linearly to create the population's allele frequencies [67]. The STRUCTURE program employs a model-based methodology that mostly adheres to the Bayesian technique of probabilistic statistics and processes data using the Markov Chain Monte Carlo (MCMC) algorithm. The identification of the relevant subpopulations and probabilistic assignment of individuals to these populations is one approach to study population structure, the other way is the likelihood approach. A model with K populations (split statistically or biologically) and a list of allele frequencies at each locus serves as the foundation for the Bayesian clustering approach [68]. This model simultaneously calculates the population's allele frequencies and divides the population into various subpopulations depending on allele frequencies and variations. To be more specific, the precise allele frequencies at each of the K populations and admixing levels for each individual animal are determined using the MCMC method of Bayesian statistics. This technique/software can be used with other kinds of markers, such as microsatellites, SNPs, *etc*. [69].

## 9 NON-PARAMETRIC APPROACHES

Non-parametric tests do not need the data modality to infer population structure. A variety of multivariate statistical analysis features are used in non-parametric tests. The two primary approaches employed in these tactics are Principal Component Analysis (PCA) and Cluster Analysis [70, 71]. These methods aim to categorize the population based on how the multidimensional diversity in genotypic data behaves linearly. These methods aid in assessing whether groupings of genotypic data represent different populations or breeds [52]. PCA seeks to minimize the number of dimensions in complicated datasets linearly. The initial vector of correlated variables is transformed into a vector of uncorrelated principal components using this dimensionality change. The primary portion of variance between populations and among individuals is determined by these fundamental components [72]. PCA aids in the analysis of various principal components for a group of markers in various livestock populations in population genetics investigations. PCA also aids in identifying populations with various components that account for the majority of the observed changes. PCA is a popular statistical method for analyzing the genetic makeup of populations. One of the key components of nonparametric approaches performed is the clustering of members of the population into different clusters based on their respective allele frequencies at various SNP variant sites. Overall, in genetics and breeding, PCA can be used to study genetic diversity, population structure, and relationships among individuals. It can also assist in identifying outlier individuals, detecting genetic anomalies, and informing breeding decisions. Finding populations that represent various population groups in the dataset is the ultimate goal of cluster analysis [73].

Non-parametric tests do not make assumptions about the distribution of the data and are used when the data are not normally distributed or when dealing with categorical or ordinal data [74]. These methods, like rank-based tests or permutation tests, can help in analyzing genetic associations, assessing genetic diversity, and identifying markers or traits under selection [75, 76]. On the contrary, parametric tests assume that the data follow a specific distribution (usually normal) and involve estimating the parameters of this distribution [77]. Parametric methods, like linear models or mixed models, are used to estimate genetic parameters, calculate breeding values, and predict genetic responses to selection [78]. These approaches provide valuable insights into the genetic architecture of traits and help in making informed breeding decisions.

## 10 DIFFERENTIATION OF RECENT AND DISTANT ADMIXTURE

Migration is an extremely potent evolutionary force. Individuals that have been admixed are the outcome of gene flow between populations. Knowing the patterns of gene flow is critical for understanding population evolution. With the elimination of mutation, the chunks provided from each parental population are assumed to be directly associated and traceable to one of the ancestors in the near hybrid generations. When two populations interbreed, a mosaic of these chunks is formed, but the chunks from the parental populations remain intricate, even if their size is altered [79]. Recent hybrids are likely to have mostly unworn ancestral haplotypes, whereas distant admixture is expected to have mostly torn-out haplotypes. This worn-out process of chromosomal chunks/haplotypes is due to genetic processes like recombination, reciprocal recombination, genetic drift, and mutation. Genetic features, for example, changes in recombination rates among chromosomal regions, create challenges for estimating time empirically from admixture data. Inferencing the recent and distant admixture may differentiate among various phylogeographic concepts [80]. Several methodologies in order to know the time of admixture are *ROLLOFF*, which looks at pairs of SNPs to see how admixture-related LD reduces with genetic distance, calculates the association between a (signed) LD statistic between two markers and a weight that reflects their allele frequency differential in ancestral populations and estimates the date by evaluating the correlation between pairs of markers as their genetic distance increases and fitting an exponential distribution using least squares [81]. Wavelet-based approach works in two parts [82]. The first is a PCA extension known as StepPCO, which extracts admixture from the genome, and the second is based on wavelet decomposition of admixture to infer the date of the mixing event. In accordance with linkage disequilibrium, a study computed the rate of LD decay at 10% frequency at places in the genome that carry derived alleles in both the ancestral and tested populations, and this approach expands the number of locations that provide information regarding timing of the admixing [83]. MALDER approach evaluates the rapid decay of admixture-induced LD in the target population while accounting for repeated admixture events in populations with relatively small sample size and the same level of admixing [84]. Scaled Block Size works by considering the ancestral population and alternate ancestry as the parental and introgressed genome, respectively [85]. The introgressed genome is used to calculate SBS by estimating the median block size of the introgressed genome as a percentage of each individual's chromosome, and the size of introgressed genome blocks is expected to be significantly linked with the period since introgression when the median introgressed block size is divided by the total percentage of the introgressed ancestor’s genome. GLOBETROTTER employs PCA as it yields (K-1) significant eigenvectors from admixture between K unique source populations and tested for three or more admixing populations by looking at two or more such eigenvectors (*p*<0.05) [86]. It can reverse the admixture process to enhance the precision of results. Parental Admixture Proportion Inference examines unphased local ancestry tracts and is made up of two parts: A model that employs genome-wide ancestry portions to predict parental admixture proportions and a Hidden Markov model (HMM) that determines admixture time frame by considering tract lengths [87]. The below table shows some of the work done using different software.

## 11 ADMIXTURE MAPPING

**Fig. (2):**
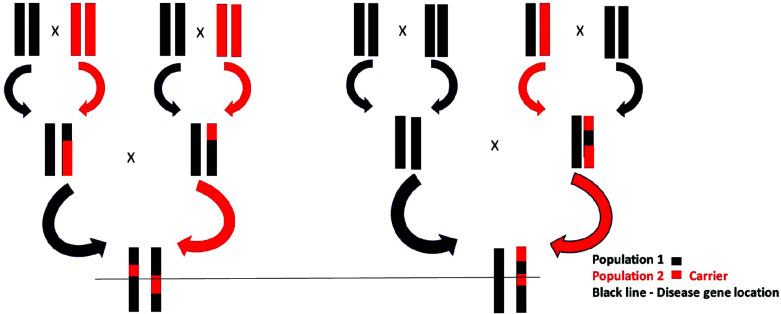
Admixture mapping of disease alleles in admixed individuals, assuming that population (red) carrying predisposed allele for the disease. Admixture mapping utilizes genetic analysis in mixed populations to identify disease-associated alleles, leveraging the linkage disequilibrium between loci. It relies on factors such as ancestral locus magnitude and time since admixture, offering a robust method with benefits including lower marker requirements and reduced susceptibility to allelic diversity.

Admixture mapping is a sort of statistical analysis in which genes are mapped using admixed populations (those created *via* gene transfer between more than one genetically diverse individual) (Fig. **2**). The strength of AM arises from the fact that linkage disequilibrium is produced between all linked and unlinked loci. According to Chakraborty *et al.*, the degree of Admixture Linkage Disequilibrium (ALD) in an admixing population is influenced by the allele frequency differences between parental populations, admixing level, dynamics of admixing, the time elapsed since admixing, and the rate of recombination between the loci [88]. ALD between linked markers degenerates more slowly than between unlinked markers, which decays more quickly (within two to four generations). The ability to distinguish between ALD produced at loci with no genetic connection and ALD generated at markers is made possible by the exponential decline in ALD with genetic distance. As a result, admixture mapping should be able to pinpoint the loci containing these alleles if the parental populations differ in a characteristic or disease due to variable frequencies of risk alleles. There are two main factors on which admixture mapping studies depend: the extent of the magnitude associated with locus ancestry, which could be assessed based on ancestry-risk ratio (proportion of risk in individuals who have two copies of a gene compared to the risk in individuals having no gene copies) and the number of generations that have passed since admixing, which could be evaluated using marker information from admixed populations [89, 90]. The key benefits of admixture mapping are that it only needs 2000–3000 ancestry-informative markers for the complete genome, and it is less prone to allelic diversity. Admixture mapping can be carried out using the Bayesian methodology and Classic Likelihood Based method [91, 92]. The prior one relies on a probabilistic framework in which K subpopulations contribute to the admixed population's gene pool, and ancestry variation on every gamete is produced *via* K distinct poisson arrival processes, whereas the second does not account for model parameter uncertainty such as allele frequencies or hierarchical reliance of individual-level factors on population-level parameters. So, the Bayesian approach is a commonly used approach for admixture mapping, and some of the Bayesian programs are ANCESTRYMAP, ADMIXMAP, and STRUCTURE [63, 93, 94].

## 12 LOCAL ANCESTRY

**Fig. (3):**
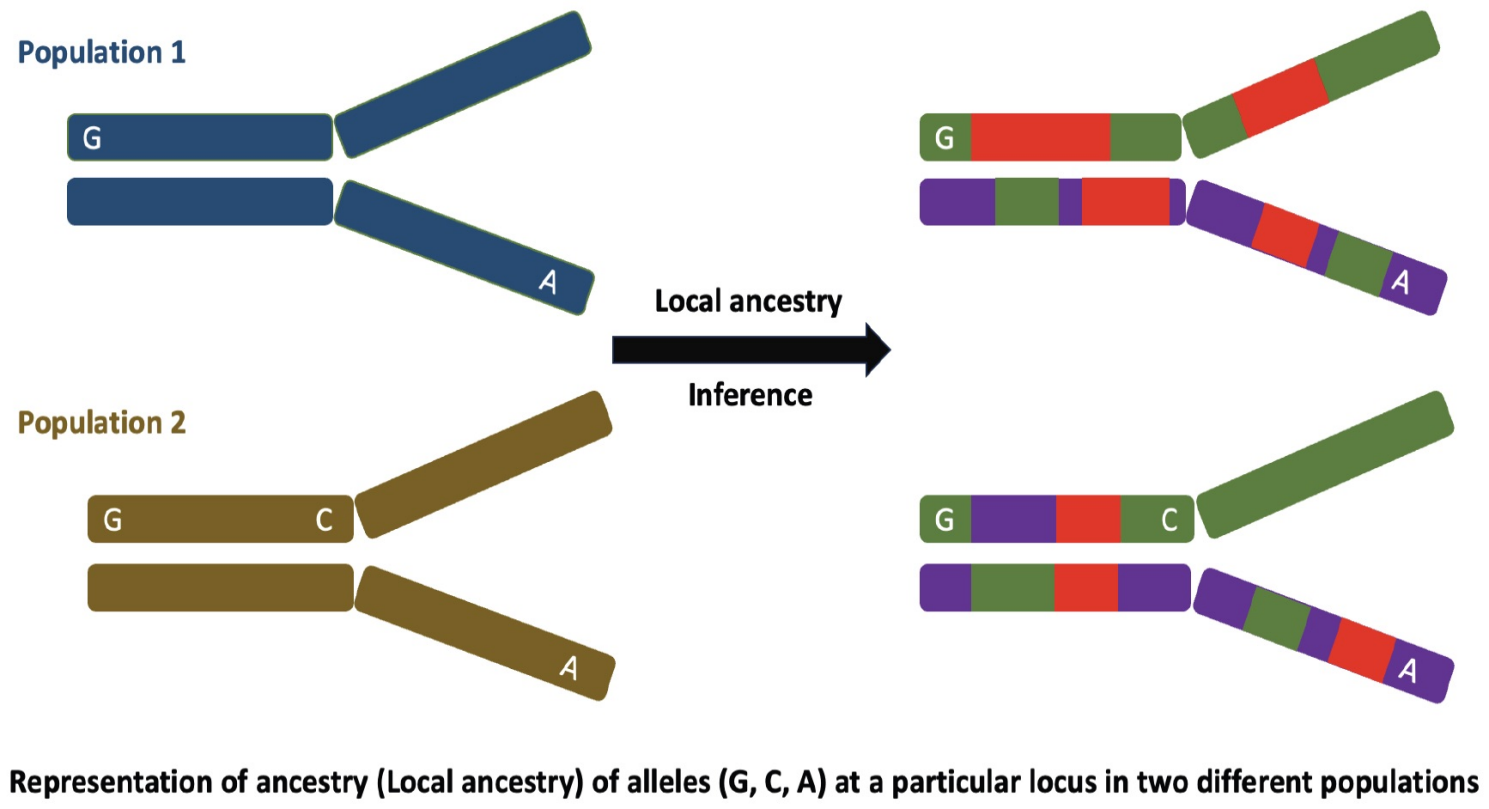
Schematic representation of local ancestry inference from admixed populations. The estimation of global genetic ancestry assumes uniform ancestry ratios across genomic loci, but limited variation at individual loci leads to divergence between local and overall ancestry. Admixture variations, influenced by genetic drift, gene flow, and selection, shape local ancestry discrepancies, provides insights into contemporary selective pressures in admixed populations.

The global estimation of genetic ancestry relies upon the assumption that each unique individual shares identical genetic ancestry ratios at each genomic locus [3]. However, it is worth noting that the potential ancestral configurations are notably constrained at a single locus, taking into account the two alleles present, resulting in limited variation, ranging from 0 to 1, irrespective of an individual's ancestral background. As a consequence, these restricted possibilities at the locus level contribute to variations in admixture proportions across different loci, leading to a divergence between the local ancestry and the overall global ancestry of admixed individuals (Fig. **3**) [95]. These variations in admixture predictions primarily arise from biological factors, including genetic drift, gene flow, and selection [3, 96-99]. It is essential to acknowledge that selection exclusively targets functional elements within specific gene regions, in contrast to genetic drift and gene flow, which impact the entire genome [100, 101]. Therefore, after a few generations of admixture, alleles favored by selection are expected to exhibit higher frequencies, leading to deviations in local ancestry from the genome-wide average [102]. These variations, whether in excess or deficiency, within the genomes of admixed individuals can be employed to identify indications of contemporary selective pressures. As the effects of specific genomic locations accumulate over multiple generations, they can be interpreted as markers of selection following the process of admixture [96]. Genetic drift, often referred to as 'noise', should be acknowledged as a factor influencing local ancestry because it can introduce significant disparities in local ancestry following admixture [103, 104]. The subsequent section will elaborate on various tools and methodologies employed for the inference of local ancestry in admixed populations.

## 13 LAMP

This approach was based on finding the Hidden Markov Model, or its expansions, that can be used to infer a broad range of parameters, including the precise location of recombination events. This approach uses sliding windows of adjacent SNPs and starts by figuring out the ideal window size. Then, it employs a clustering technique that uses these windows to determine each individual lineage. The most likely ancestral populations at each SNP are then determined by a majority vote across all windows that coincide with the SNP. This straightforward strategy offers several benefits. In the beginning, it demonstrates analytically that the algorithm's estimates are asymptotically accurate across the entire genome. Second, it optimizes fewer parameters than other techniques, making the optimization quicker and more reliable. Thirdly, it also takes the advantage of not requiring ancestral genotypes to infer locus-specific ancestries, in contrast to other approaches. Fourthly, its accuracy improves by increasing the quantity of the reference data due to its efficiency in handling big reference sets. Finally, its algorithm successfully converts the larger reference set and reduces divergence between the reference and ancestor populations with superior accuracy [105]. The major drawback of LAMP is that it necessitates the usage of a physical map as well as statistical characteristics, such as the Hidden Markov model's hidden state count and window size that is utilized for assuming constant local ancestry [106]. Yougbaré *et al.* analyzed local ancestry deviations from the average for each SNP across 29 autosomes to detect potential regions under selection in trypanotolerant Baoulé cattle and their crossbreds using LAMP [101]. Significant deviations were found on chromosomes 8 and 19 in positive animals, while negative animals showed higher deviations on chromosomes 6, 19, 21, and 22, with candidate genes like PDGFRA on chromosome 6 and CDC6 on chromosome 19 associated with trypanotolerance in West African taurine cattle.

## 14 WINPOP

It is the locus-specific ancestry method that starts with the LAMP framework and counts for a single recent recombination per window. It employs a dynamic programming approach to loop through the positions of the window and determines the possibility of each point having an ancestor either upstream or downstream. Furthermore, it selects the window length individually at each point based on the local genetic difference between the two ancestral populations within that window, and in each window, it anticipates exactly one recent recombination event. This differs from LAMP's window length calculation, which is based solely on the number of iterations and recombination frequencies. It assumes that the SNPs in the data are uncorrelated and less informative SNPs are removed [107]. To quote an exemplary study using WINPOP, Yang *et al.* interrogated genome-wide germline SNP genotypes in random samples of children with acute lymphoblastic leukemia (ALL) and observed that the genomic variations associated with Native American ancestry were responsible for the risk of relapse of ALL [108].

## 15 HAPMIX

This methodology uses phased data from unadmixed reference populations, which are genetically identical to the actual ancestral populations. HAPMIX posits that the admixed population under study resulted from the mixing of two ancestral populations. Although mistakes could theoretically result from differences between the reference populations and the true ancestral populations, in practice, HAPMIX is resilient to this problem in a number of realistic scenarios [109]. The main idea behind this method is to view each of the admixed individual haplotypes as samples taken from the reference populations. HAPMIX calculates the probability that a haplotype from an admixed person will be a better statistical match to one or both reference populations at each location in the genome. These likelihoods are combined with data from nearby loci using HMM, which yields a stochastic prediction of ancestry at every locus. Two-scales transition can take place using this method. Small-scale transitions often occur every few tens of thousands of bases between haplotypes from a reference population, and for a newly admixed population, the large-scale transitions can involve up to tens of millions of bases between the reference populations. The primary flaw of this methodology is that it requires specifications of various biological characteristics such as genomic maps, rate of mutations and recombinations, average ancestry coefficients, and an average number of iterations since admixing, a lot of time for processing and only takes into account two ancestral populations at once. HAPMIX is employed to detect ancestral chromosomal segments in Romani population genomes. Interestingly, various Romani populations from Central Europe (Slovakia, Hungary, and Romania) and the Balkan region (Bulgaria and Croatia) exhibit low mean values of genetic admixture, suggesting that the European dispersion of the Romani people occurred predominantly through the Balkans after a swift migration with moderate gene flow from the Near or Middle East [111].

## 16 PCAadmix

PCA is a quick, nonparametric technique for finding structure in data. It distinguishes the main axes of ancestry when applied to genetic data and divides samples according to population genetic structure. It is easier to deconvolve ancestry tracts in admixed individuals since the admixed individuals are displayed between the ancestral populations. Since the positions of admixed people in relation to groups of ancestral individuals provide interpretation rather than the PCs, it may not be as interpretable as admixture models. So in order to avoid this flaw, PCs are expanded to PCAadmix, which uses PCA to assign higher weights to variation, which provides more ancestry-related information. This approach utilizes phased data, shorter windows of SNPs and an HMM to probabilistically represent each window's ancestry and infer the ancestry proportions of individual parents on each chromosome [112]. In a notable study utilizing PCAdmix, Spangenberg *et al.* identified chromosomal segments of Amerindian descent, indicating the existence of indigenous genetic ancestry in present-day descendants [113]. The Charrúas, an Amerindian group that lived in Uruguay during the period of European colonial contact, were found to possess specific haplotypes that were abundant among them but rare in other studied Amerindian groups.

## 17 SupportMix

It is a machine-learning technique that has a two-stage approach in which the initial stage employs support vector machines (SVM), a subcategory of supervised machine learning algorithms, that determines putative ancestors of genomic regions. SVM was acknowledged as one of the most effective approaches for generic classification tasks in order to detect transitions between parental sources in admixed genomes. The second level adopts a refining technique based on the Hidden Markov Model (HMM). It is a reliable strategy that could be scaled to a genome-wide analysis by taking into account more than fifty parental populations. It can undertake analysis by looking at numerous populations from around the world simultaneously as potential ancestors without being worried about how they relate to the target group and will return to the population that is genetically closest to the ancestral population. The major advantage is that it is more precise but also resilient to changes in the parameter [114]. To quote an example, SupportMix analyzed the ancestry of the Qatar population using 55 world populations from the Human Genome Diversity Panel, revealing detailed insights into the region's genetic history. It confirmed the presence of three major sub-populations in Qatar with mainly Arabic, Persian, and African ancestry. Additionally, SupportMix identified that the Persian group's ancestry is more closely related to populations from Greater Persia rather than China and the African group's ancestry is of sub-Saharan origin rather than Southern African Bantu origin, as previously believed [115].

## 18 ChromoPainter

It is the best way to infer ancestry when there is a problem or a lack of data for the admixed population, as it uses information from huge panel data that is even unrelated to the targeted admixed population. Both current and historical populations’ worldwide ancestry can be reconstructed using this technique, and simulation-based comparisons have demonstrated that it has high accuracy at the genome-wide level, even when just a few reference samples are available. Later, it utilizes Nonnegative Least Squares to deduce the painting information [116]. As an example study to quote for this tool, Kumar *et al.* conducted a study on 110 Roman Catholics from three different locations on the West Coast of India to investigate their genetic history [117]. They discovered that Roman Catholics exhibit a strong genetic affinity with Indo-European linguistic groups, especially Brahmins. The study also detected genetic signals of Jewish ancestry in Roman Catholics through linkage disequilibrium-based admixture analysis, a signal not found in other Indo-European populations in the same geographical regions. Additionally, the analysis indicated that Roman Catholics have a distinct South Asian-specific ancestry and have undergone significant genetic drift.

## 19 RFMix

It is a discriminatory strategy for modeling ancestry across a haplotype sequence of admixed individuals of known or assumed ancestry. Such strategies directly describe the reliance of unseen factors (such as ancestries) on observable factors (such as alleles). In this technique, a Conditional Random Field (CRF) generated by random forest models learned on reference panels are used to estimate local ancestry inside every window of each chromosome. Following the assignment of ancestries to windows in admixed chromosomes, it utilizes them to increase inference accuracy by employing an expectation-maximization (EM) step to better understand haplotype trends in parental populations. Estimation of ancestry based on RFMix is more precise and faster compared to various techniques, such as LAMP (approximately 33-fold faster) and SupportMix (about 1.7-fold faster) [118]. Daya *et al.* conducted admixture mapping in the South African Coloured population using RFMix to identify novel tuberculosis susceptibility genomic regions [119]. They identified several promising regions associated with San ancestry and African ancestry, notably on chromosomes 15q15 and 17q22, which are near genomic regions previously linked to tuberculosis. The study also highlighted immune-related susceptibility genes like GADD45A, OSM, and B7-H5 in these identified regions.

## 20 EILA

Efficient Inference of Local Ancestry in admixed individuals is based on three phases to address the methodological issues. In the initial stage, genotypes in admixed individuals are given a number score (with a range of 0-1) to better quantify how closely related the SNPs are to a particular ancestral group. In the second stage, the breakpoints of the ancestral haplotypes are determined using fused quantile regression, and in the third stage, the k-means classifier is employed to infer ancestry at each site. The main advantage of EILA is that it relaxes the requirement of linkage equilibrium and employs all genotyped SNPs rather than just unlinked loci to boost the power of inference [115]. In an analysis of the Singaporean chicken population using EILA, it was revealed to be highly diverse, with red junglefowl-introgressed alleles ranging from 5% to 97%. The study inferred that genes selected for domestication in this population, such as SLTM, CFAP97, CAPS2, C2CD5, and DYNC2H1, originated from red junglefowl ancestry [120].

## 21 ASPCA

The purpose of the Ancestry-Specific PCA approach is to determine the subcontinental origin of haplotypes across the entire genome, offering an improved understanding of the ancestors. It also examines tract length patterns of genomic regions related to different origins to analyze biological models of modern demographic evolution since the advent of intercontinental mingling [116]. Lucas-Sánchez *et al.* used ASPCA to examine the genetic impact of trans-Saharan migrations in North Africa, revealing heterogeneous and generally low-frequency genomic segments of sub-Saharan origin among North Africans [121]. Two significant admixture events were identified: one around the thirteenth to fourteenth centuries CE involving North Africans and a Western-sub-Saharan-like source, and another around the seventeenth century CE involving Tunisians and an Eastern-sub-Saharan-like source. These events coincide with the peak of the trans-Saharan slave trade. The findings suggest ongoing genetic interactions between sub-Saharan and North African populations, contributing to the complex genomic composition of North Africa.

## 22 LOTER

Loter program was developed to infer local ancestry for a wide range of taxa for those whose biological parameters, such as admixing timeframes and recombination rates, are unknown. It relies on the mimicking approach established by Li and Stephens, which assumes that admixed individual haplotypes are viewed as a matrix of preexisting ancestral haplotypes in a given set of ancestral haplotypes from a potential source parental population. It involves a smoothing control value called regularization parameter (*λ*), and this value is dependent on a complex set of analytical and biological variables, involving rates of mutation and recombination and implements a process where it averages solutions for various regularization parameter (*λ*) values to avoid the challenging regularization parameter selection. It requires phased haplotypic information for both reference and target population and accounts for phase errors. This package depends on parameterized optimization problems that have a single regularization factor, which penalizes switching among ancestral haplotypes and is used to find solutions to the optimization issue, and its computing complexity scales linearly with the number of markers and individuals from the initial populations. It estimates the time of admixture using restored ancestry sequences, and the results are accurate in terms of the length of time frame since admixing happened. This method's key benefits for determining local ancestry are that no genetic maps are needed, there are no restrictions on the number of SNPs, and admixture time is not necessary [118]. The analysis using LOTER by Wedger *et al.* revealed significant insights into the genomic consequences of crop-weed hybridization and selection for herbicide resistance in contemporary weedy rice populations. The results showed a clear bias toward evolving back to their weedy ancestor, with most contemporary weeds being crop-weed hybrid derivatives. The genomes of these hybrids have evolved to be more like their weedy ancestors, indicating a shift in population dynamics. Haplotype analysis demonstrated extensive adaptive introgression of cultivated alleles at the resistance gene ALS, suggesting that selection pressure favored these alleles [122].

## 23 FLARE

Fast local ancestry estimation uses an enhanced model to achieve high accuracy, and it incorporates computational methods created for genotype imputation to obtain remarkable computing performance and the usage of composite reference haplotypes speeds up computation [107]. It can be utilized for datasets containing tens of thousands of sequenced individuals and deliver higher accuracy on massive amounts of data [123]. In one of the studies using FLARE software, the Andean cohort's ancestry was analyzed, revealing a small fraction resembling the component identified in an Iberian population from Spain (IBS) and a large non-IBS, likely Native American component. Additionally, FLARE was used to determine local ancestry at the EPAS1 gene region plus 100 kb up- and downstream of the EPAS1 gene using the Thousand Genome Project phase 3 as the reference population. The results showed no significant admixture at this locus. These findings suggest that the Andean cohort has a unique genetic ancestry, with a mixture of Iberian and Native American components, and that the EPAS1 gene region has not experienced significant admixture, indicating a potential role for the EPAS1 gene in high-altitude adaptation in Andean highlanders [124].

## 24 SALAI-Net

The Species-Agnostic Local Ancestry Inference Network is a two-stage method that begins with a source comparing layer, which offers window-level first estimations, followed by a smoother layer that improves the initial projections by leveraging adjacent window data as well as minimizing the shortcomings. After being familiar with particular conditions, SALAI-Net can be utilized for local ancestry inference across any other species or for any group of ancestries without the requirement for further tuning or retraining [125]. The SALAI-Net method was applied to three different datasets by Sabat *et al.*: whole-genome human sequences, human genotyping array samples, and whole-genome sequences from dogs [125]. The method outperformed previous approaches in terms of balanced accuracy and demonstrated the ability to generalize between different species, chromosomes, and datasets. When tested on human data, SALAI-Net showed improved performance and speed compared to existing methods, even when trained on human data and applied to dog breeds. The results suggest that SALAI-Net is a versatile and efficient method for local ancestry inference, applicable to a wide range of species and ancestry groups without the need for retraining or biological parameters.

## 25 BCSYS (LOCAL ANCESTRY CLASSIFIER)

It is more computationally efficient and enables us to use a larger breed DNA reference panel. Large reference panels, in turn, allow for more breeds to be called and for increased accuracy due to the inclusion of more reference samples per breed. Furthermore, the BCSYS algorithm was specifically tuned to improve accuracy for mixed-breed samples. Finally, unlike our legacy algorithm, BCSYS is a local ancestry classifier, which means that in addition to calling the total proportion of breeds throughout an animal’s genome, it also assigns ancestry labels to very specific small segments of chromosomes. One new feature is that the local ancestry results are now used to train a machine learning model that predicts the purebred status of an animal. However, the local ancestry classifier will also drive future product development, detailing how an animal’s physical traits relate to their individual ancestry [126]. The BCSYS Local Ancestry Classifier algorithm was used in the study to determine breed assignment based on comparison to a reference panel of over 21,000 dogs of known ancestry from more than 50 countries [127]. The algorithm classified dogs as purebred if they had 90% or greater single-origin ancestry, and for breed-specific analyses, the threshold was lowered to 80% or greater single-origin ancestry to obtain larger cohorts for analysis. This approach allowed for the identification of breed-specific risk factors for cherry eye. The analysis revealed that certain breeds were at higher risk for cherry eye, including the Neapolitan Mastiff, English and French Bulldogs, Cane Corso, Lhasa Apso, and American Cocker Spaniel. These findings suggested the importance of breed-specific genetic factors in the development of cherry eye and hence highlight the importance of the BCSYS tool in population genetics.

## 26 AFA (ANCESTRY-SPECIFIC ALLELE FREQUENCY)

It estimates the frequencies of biallelic variants in admixed populations with an unlimited number of ancestries. It uses maximum-likelihood estimation by modeling the conditional probability of having an allele given the proportions of genetic ancestries. It is applied using either local ancestry interval proportions encompassing the variant (local-ancestry-specific allele frequency estimations in admixed populations) or global proportions of genetic ancestries (global-ancestry-specific allele frequency estimations in admixed populations), which are easier to compute and are more widely available [128]. The AFA tool was used in the study to estimate the frequencies of bi-allelic variants in the admixed Hispanic/Latino population based on global proportions of genetic ancestries [128]. The tool identified Amerindian-enriched variants with frequencies of at least 5% in Amerindian ancestry and less than 1% in African and European ancestries. Similarly, African-enriched variants were identified with frequencies meeting specific criteria. Upon annotation of ancestry-enriched variants, the APOE-ɛ4 gene, having a mild cognitive impairment (MCI), was identified. This highlights the importance of the AFA tool in eugenics-related studies (Tables **1** and **2**).

## 27 APPLICATION OF ADMIXTURE ANALYSIS

### 27.1 Implications of Admixture Analysis in Pigmentation

Recent genetic studies have delved into both normal and pathological variations in pigmentation [129]. Some of these inquiries have showcased the ability to predict color phenotypes based on genotype data, demonstrating varying levels of accuracy. This emphasizes the significance of such studies, especially in the case of forensic practices [130]. Furthermore, it has been suggested that specific genetic variants linked to pigmentation might influence susceptibility or resistance to skin cancer [131]. This association is attributed to the adaptation of different skin tones to diverse environments following the migration of anatomically modern Homo sapiens from Africa to other continents [132]. While the effectiveness of utilizing admixed subjects for gene detection has been recognized for several decades [88]. It is only in recent years, aided by high-throughput SNP genotyping, that the full potential of this approach has been revealed [22, 133]. The extensive human diaspora resulting from historical events, such as the European colonization of the Americas during the age of exploration, has given rise to the establishment of admixed populations that have persisted for centuries and are now available for research. For example, a comprehensive genome-wide investigation of African–American patients with chronic kidney/end-stage kidney disease has presented compelling evidence linking the *MHY9* gene to an increased predisposition to the condition associated with African ancestry [134]. Moreover, these studies have documented the extensive diversity in the genotype-phenotype architecture of pigmentation across various human populations. For instance, a study demonstrated that in addition to the classical genes *SLC24A5* and *SLC45A2*, others, such as *OPRM1* and *EGFR,* have also played a role in the differences in pigmentation between Native Americans and Europeans [129]. Furthermore, Norton *et al.* suggested that polymorphisms in *SLC24A5, SLC45A2,* and *TYR* predominantly contribute to the evolution of lighter skin color in Europeans but not in East Asians [135]. This indicates the recent convergent evolution of lighter pigmentation phenotypes and emphasizes the importance of natural selection in this process. In an admixed population, the influence of individual loci on a quantitative trait can be identified by observing either a correlation between genotype and phenotype or a correlation between local ancestry and phenotype [24]. Genotype-based approaches are expected to be more effective for traits where the causative allele exists at similar frequencies in ancestral populations. On the other hand, ancestry-based approaches are likely to be more powerful for traits where the causative allele displays significant frequency differences across ancestral populations [136].

### 27.2 Population Admixture in Forensics

Population admixture is a prevailing feature of populations on continental margins and has been a recurring phenomenon since the initial migration of small human groups. Over the course of 2500 years, populations have increasingly interacted through trade, conquest, and slavery [137]. The past two centuries of urbanization and mass movement have dismantled cultural and social barriers that previously substituted for geographical separation. Consequently, forensic ancestry analyses are likely to reveal a significant proportion of admixture patterns among tested individuals. Investigators are particularly intrigued by admixture as it hints at the possibility of unique combinations of physical characteristics in a suspect. In a specific case, the *MC1R* gene in a DNA sample, indicating predominantly African co-ancestry along with an *MC1R* V60L ‘r’ variant (rs1805005-T), suggests a potential combination of red hair and dark skin [138]. Therefore, it is valuable to evaluate how the three outlined analytical approaches (Bayes, PCA and STRUCTURE) to forensic ancestry inference handle admixture. Establishing a suitable detection framework can prompt subsequent tests to enhance the genetic differentiation of contributor populations, thereby improving the estimation of co-ancestry components, especially with the addition of Y and mtDNA data [139, 140]. In the realm of biogeographic ancestry (BGA) inference from forensic DNA, there have been notable advancements. Here, recently introduced forensic BGA tools are discussed, encompassing marker selection, genotyping multiplex design, and the statistical analysis of resultant data. The selection of Ancestry-Informative DNA markers (AIMs) involves assembling a suitable panel tailored for a specific set of population differentiations. The subsequent statistical approach applied to the genotype data should not only predict BGA using reference population datasets but also possess the capability to discern co-ancestry in individuals with mixed backgrounds. As the precision of BGA inferences from DNA largely hinges on the number of AIMs employed and targeted Massively Parallel Sequencing (MPS) holds the most extensive multiplex capacity among current forensic DNA technologies, the focus here is specifically on forensic BGA tools relying exclusively on targeted MPS. Recent developments in these tools have predominantly centered around autosomal Single Nucleotide Polymorphisms (SNPs) as the preferred AIMs [141]. However, there is a growing interest in the ancestry informativeness of autosomal micro haplotypes (MHs), which involve combinations of closely situated SNPs in short sequences easily detected through single-strand sequencing with MPS.

Given our focus on bi-parental BGA inferred with autosomal AIMs, mention of autosomal Short Tandem Repeats (STRs), commonly used in forensic DNA profiling for individual genetic identification, will be made only if they are part of MPS tools concentrating on autosomal AIMs [142]. While autosomal STRs can contribute to viable population differentiations, their power is generally less than that of autosomal AIM SNPs, and STR tests have not been specifically adapted for BGA [141].

### 27.3 Post-admixture Signals of Selection (PASS) or Adaptive Admixtures

Admixed populations offer unique chances to look into recent selections. The original populations were geographically isolated before admixture, and different environments played a crucial role in the evolution of their genomes. The movement of formerly isolated groups or populations might have exposed the members of parental populations to novel environments, which may have led to changes in their adaptation or the infections to which they have been subjected. This sort of selection might be different from that experienced by static populations, where minor modifications to the environment may occur progressively, enabling the frequency of rare advantageous alleles to rise [95]. This method is used to find out ancestral or parental signatures of selection by investigating genomic areas in an admixed population that show exceptionally substantial variances within ancestry proportions relative to how it is typically observed throughout the genome. In order to find post-admixture signals of selection, it is required to compute delta ancestry (Δ ancestry), which is excess or deficiency in terms of ancestry at each SNP by utilizing admixture components as the base [95].

This methodology has been effectively utilized to identify recent selection in mixed Swiss Fleckvieh cattle and selection for Zebu-introgressed regions in Colombian creole taurine cattle [13, 134]. Yougbaré *et al.* applied a similar approach to detect significantly different local admixture levels, identifying five chromosomes with higher deviation from average ancestries, showing an excess of Baoulé ancestry potentially associated with higher tolerance to trypanosomiasis [101]. They identified regions deviating from average ancestry with a higher amount of Baoulé proportions on chromosomes 6, 8, and 19 in trypanosome-negative individuals and found higher Baoulé ancestry in chromosome 8 (35–50 Mb) also in trypanosome-positive cattle, suggesting these regions may contain beneficial Baoulé haplotypes unrelated to trypanosomosis tolerance. Noyes *et al.*identified several genes on chromosome 8, including VAV1, PIK3R5, RAC1, VAV2, GAB2, and INPP5D, to be under selection in Muturu and N’Dama cattle breeds in response to trypanosome infection [143, 144]. Ward *et al.* detected evidence for mitonuclear coevolution across hybrid African cattle populations, showing a significant increase of taurine ancestry at mitochondrially targeted nuclear genes [145].

Based on the degree of LD in admixed populations, it is necessary to calculate the thresholds of selection signals across the entire genome using numerous tests of hypotheses for correction (employing Bonferroni correction) and considering five thousand and one thousand distinct segments. Following research on human admixing by Tang *et al.*, local ancestry variations equivalent to five thousand hypotheses and concerning a thousand hypotheses were investigated. To determine the degree of importance level for the excess or deficiency of SNPs across the entire genome of admixed individuals for every pristine ancestry, permutation tests were done. Then, the local ancestry estimates from all the chromosomes for each individual were combined. Later on, the genome was cut twice at random locations, and then the two portions of the genome were rearranged for each individual separately. Assuming LD was spread uniformly throughout the genome, this kind of permutation retains the amount of LD. Then, a percentage quantile transformation step was included after implementing 20,000 permutations. In order to match corresponding spotted distributions in each permutation test, the SD of the permuted data distribution (trimmed at the conclusion of each test by 0.05) was multiplied by a scaling factor, and also, each permutation's minimum and maximum values were calculated. The maximum and minimum permutation values were utilized to define one and five percent threshold levels, which showed considerable departure of the observed local ancestries from the genome-wide average ancestry [95]. Several statistics within and between the populations can be employed to identify the signals of selection in admixed animals. The dataset initially undergoes phasing, after which overlapping selection signatures in the delta ancestry regions are discovered. The scores represent the regions of the genome that exhibit surprisingly high percentages of haplotype homozygosity among or between populations. Finally, the structural annotation and the functional annotation are used in the genomic research to identify genomic differences between the populations. In order to discover potential locations of substantial-high delta ancestry after admixing and to determine if these locations are likewise highly differentiated in the parental breeds, allele frequency differentiation values on each chromosome were calculated and averaged [13].

### 27.4 Ancestral Recombinant Graphs

**Fig. (4):**
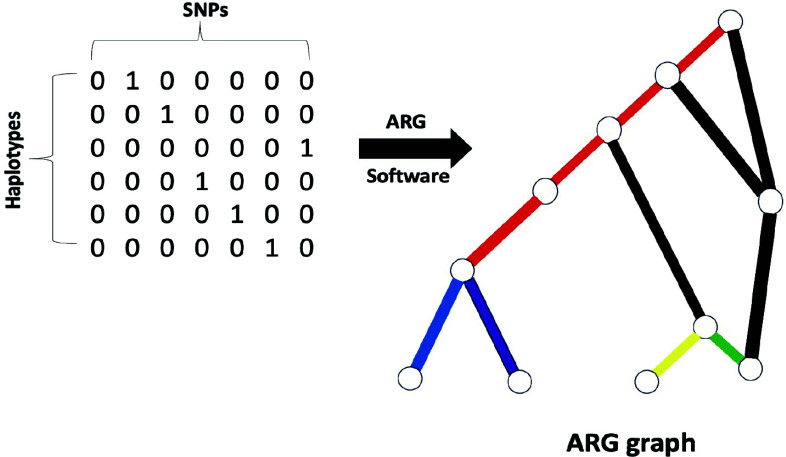
Digrammatic representation of ancestral recombination graph (ARG) construct. ARG represent the comprehensive genealogy of DNA sequences, capturing all coalescence and recombination events. Inferring ARG involves detecting recombination breakpoints, reconstructing evolutionary trees for recombinant fragments, and combining them, providing insights into genetic material transmission and facilitating mapping and imputation.

ARG can fully represent the association framework of an ensemble of collinear identical sequences of DNA [146]. It records all coalescence and recombination events that have occurred since differentiation and describes a comprehensive genealogy at each genomic location, which makes it different from that of the phylogeny inference as it does not account for recombination [147]. The standard approach for inferring an ARG consists of detecting breakpoints in recombination, then reconstructing the evolutionary tree for each recombinant fragment, and lastly, combining all reconstructed trees (Fig. **4**) [148]. As one traverses from left to right along a chromosome, the local tree remains stable until a recombination breakpoint is found. At that point, it is updated to build a new tree in the way indicated by the change in route at the corresponding recombination node in the ARG. As a result, it can be considered interchangeable with a succession of local trees and the recombination events that connect each tree to the next [149]. It provides an optimal amount of information regarding trapped genetic material that exists between two linked ancestral loci but is not passed on to any modern sample for mapping the ages and haplotypic background of mutations and also imputes missing data optimally. It is additionally feasible to calculate the TMRCA (time to the most recent common ancestor) of admixed and admixing haplotypes [150, 151].

The format is a fundamental aspect of ARG, and there have been very few ARG formats established [152]. There's a requirement for an approved format that will enable easy communication with various ARG applications. Some tools are utilized to infer ARG. *ArgML* is an XML-based standard for storing precise information on the ARG, even if numerous recombinations take place at the same inter-site link [153]. *IRiS* discovers recombination events with high confidence in their shared ancestry and combines these recombinations into an ancestral recombination network [154].; *ACG* utilizes the Bayesian MCMC approach to determine posterior distribution parameters like population size, transition to transversion ratio, recombination rate and the modified Felsenstein pruning approach to infer ARG [155] *ARGweaver* is based on the partitioning of time (in which all recombination and coalescence processes are permitted to take place at a particular set of time periods) and the Hidden Markov Model to compute ARG [156] Rent + uses more information (singletons) contained in the data, builds guide trees from haplotypes, and uses them to infer local genealogies [157-159]. DeCoSTAR reconstructs the organization of ancestral genomes or genes as a set of neighborhood relations between pairs of ancestral genes or gene domains [160]. Relate employs a haplotype-mimicking model to determine pairwise distances between samples [161]. Following that, it employs MCMC with a coalescence antecedent to deduce coalescence time on these trees. SARGE operates on phased data, does not require any previous hypotheses other than symmetry, heuristically calculates branch lengths, and minimizes inferring regarding unseen linkages by retaining polytomies in outcome [151]. ARG-Needle works on genotype or sequence data by threading one haploid sample at a time to an existing ARG iteratively [162]. KwARG is based on parsimony that finds credible genealogical histories with a minimum or near-minimum number of hypothesized recombination and mutation events (Table **3**) [163].

### 27.5 Increasing Heterosis

This application is a novel application of local ancestry analysis. Heterosis, also known as hybrid vigor, is a phenomenon where the offspring of two different purebred lines have superior characteristics to their parents [165]. Utilization of heterosis is the exclusive goal of crossbreeding. The heterosis in the crossbred population is explained by the dominance theory, which postulates that the parental lines are homozygous dominant for different loci – when crossed, produce progeny with the dominant gene at all loci [166]. Overdominance theory postulates that the heterozygote is superior to either homozygote (parents), and epistasis theory postulates that gene interactions are responsible for heterosis [165, 166]. Since epistasis of the gene is also cited as one of the reasons for heterosis, we propose that a cross with a better combination of genes/polymorphisms can be identified with the local ancestry and retained in the herd, and a cross carrying the inferior combinations of the genes can be culled in the early age [134, 136]. Since heterosis is measured as “Heterosis (H) = [ (Mean of F1 offspring) - (Mean of parents) /Mean of Parents ] x 100”, retaining better crosses will increase the mean of F1 offspring, and hence it will increase the overall heterosis.

However, it is crucial to note that while local ancestry estimation can indeed aid in identifying favorable gene combinations, the practical implementation may encounter complexities. Factors like environmental interactions, genetic drift, and the multifaceted nature of traits can influence outcomes. Additionally, rigorous validation and accurate estimation methods are essential to ensure the reliability of local ancestry estimates.

## CONCLUSION

Admixture analysis can be used to estimate inheritance levels from different source populations in an admixed population, and based on that, better breeding decisions can be made. Local ancestry can be exploited in tracking the inheritance of particular chunks of haplotype, and thus better combinations of haplotypes can be retained, and post-admixture selection signatures frequencies can be increased or transferred to the population of choice to improve overall fitness in a specified production system.

## AUTHORS' CONTRIBUTIONS

All authors contributed to the study's conception and design. Material preparation and data collection were performed by RCG, KGC, PR, NS, KKK, CSC, and OML. The first draft of the manuscript was written by RCG, KKK, IG, SS, and SPD, and all authors commented on the previous versions of the manuscript. All authors read and approved the final manuscript.

## Figures and Tables

**Table 1:** Brief description of several methods for local ancestry estimation.

**Software**	** Algorithm **	**Number of Ancestral Population**	**Phasing of Data**	**Genetic Map**	**Limitation of the Number of SNPs**	**Admixture Time Required**	**References**
LAMP	Clustering and HMM	2,3,5	NO	NO	NO	NO	[104]
HAPMIX	HMM	2	NO	YES	NO	YES	[110]
RFMix	CRF	≥ 2	YES	YES	NO	YES	[118]
LOTER	Single Layer HMM	≥ 2	YES	NO	NO	NO	[107]
PCAdmix	HMM and Local PCA	≥ 2	YES	YES/NO	NO	NO	[112]
WINPOP	Clustering and HMM	2,3,5	NO	NO	NO	NO	[107]
Support Mix	SVM	≥ 2	YES	YES	NO	YES	[114]
Chromopainter	HMM	≥ 2	YES	YES/NO	NO	NO	[116]
EILA	k-means	2 or 3	NO	NO	NO	NO	[115]
ASPCA	ssPCA	≥ 2	YES	NO	NO	YES	[116]
FLARE	HMM	2 or 3	YES	NO	NO	NO	[123]
BCSYS	HMM	≥ 2	YES	YES	NO	YES	[126]
AFA	HMM	≥ 2	NO	NO	NO	NO	[128]

**Table 2:** Scope and limitations of different methods for local ancestry estimation.

**S. No.**	**Name of the Software**	**Scope**	**Limitations**
1.	**LAMP** [104]	**Analytical Accuracy**: Accurate across the entire genome. **Parameter Optimization:** LAMP optimizes fewer parameters than other techniques, leading to less computational time **No Need for Ancestral Genotypes:** LAMP does not require ancestral genotypes to infer locus-specific ancestries, unlike other approaches. **Scalability:** The accuracy improves with larger reference data sets, making it efficient for handling big reference sets. **Reduced Divergence:** LAMP can reduce divergence between reference and ancestor populations with superior accuracy.	**Physical Map Requirement:** LAMP necessitates the use of a physical map. **Statistical Characteristics:** It requires statistical characteristics such as the hidden state count and window size from the Hidden Markov model for assuming constant local ancestry.
2.	**WINPOP** [107]	**Single Recombination Events:** WINPOP is designed to identify and account for single recent recombination events per window, providing a more detailed analysis of local ancestry. **Dynamic Window Length Selection:** WINPOP dynamically selects the window length at each point based on the local genetic differences, allowing for more accurate inference in regions with varying levels of admixture. **Efficient Handling of SNP Data:** By assuming uncorrelated SNPs and removing less informative ones, WINPOP can effectively handle large SNP datasets, making it suitable for analyzing genetic data from diverse populations. **Extension of LAMP Framework:** WINPOP builds upon the LAMP framework, which has been shown to be effective in inferring local ancestry, providing a solid foundation for its methodology.	**Assumption of Uncorrelated SNPs:** WINPOP assumes that SNPs in the dataset are uncorrelated, which may not always hold true in practice and could affect the accuracy of the inference. **Limited to Single Recombination Events:** While WINPOP's focus on single recombination events per window provides detailed analysis, it may not be suitable for detecting multiple or complex recombination events within a window.
3.	**HAPMIX** [110]	**Phased Data Usage:** HAPMIX utilizes phased data from unadmixed reference populations that closely resemble the true ancestral populations of the admixed group, allowing for accurate ancestry inference. **Admixture Modeling:** The methodology assumes that the admixed population under study is a result of mixing between two ancestral populations, enabling it to model admixture scenarios effectively. **Haplotype Matching:** By treating each haplotype of an admixed individual as a sample from the reference populations, HAPMIX can calculate the probability of each haplotype being a better match to one or both reference populations at each genomic location. **Hidden Markov Model (HMM):** HAPMIX combines these probabilities with data from nearby loci using an HMM, providing a stochastic prediction of ancestry at every locus. **Transition Modeling:** It can model both small-scale and large-scale transitions between haplotypes from reference populations, capturing the complex admixture patterns that may occur in the population.	**Biological Characteristic Requirements:** HAPMIX requires accurate specifications of various biological characteristics such as genomic maps, rates of mutations and recombinations, average ancestry coefficients, and an average number of iterations since admixing, which can be challenging to determine and may introduce errors. **Two Ancestral Populations Limitation:** HAPMIX only considers two ancestral populations at a time, which may not fully capture the complexity of admixture in some populations that have more than two ancestral components.
4.	**PCAadmix** [112]	**PCA Extension:** It extends Principal Component Analysis (PCA) to improve the interpretability of results and enhance the resolution of ancestry inference, particularly in regions with complex admixture patterns. **Utilization of Phased Data:** By using phased data, PCAadmix can better capture the haplotype structure. **Short SNP Windows:** The use of shorter windows of SNPs allows for a more detailed analysis of genetic variation and ancestry. **Probabilistic Ancestry Representation**: PCAadmix utilizes a Hidden Markov Model (HMM) to probabilistically represent ancestry at each window, providing a more nuanced understanding of ancestry proportions.	**Genetic Map Requirements**: Genetic maps for most of the livestock are not available. **Admixture Modeling Limitations:** PCAadmix, like other methods, assumes a specific model of admixture (mixing of two ancestral populations), which may not fully capture the complexity of admixture patterns in all population.
5.	**SupportMix** [114]	**Machine Learning Approach**: It uses a two-stage approach, with the initial stage employing Support Vector Machines (SVM), a type of supervised machine learning algorithm, to detect transitions between parental sources in admixed genomes. **Refining Technique:** The second stage of SupportMix uses a Hidden Markov Model (HMM) as a refining technique to further improve the accuracy of ancestry inference. **Scalability:** SupportMix is scalable to genome-wide analysis, as it can consider more than fifty parental populations simultaneously as potential ancestors. **Genetic Distance Consideration:** It considers genetic distances to determine the population genetically closest to the ancestral population, providing more precise ancestry inference. **Parameter Resilience:** SupportMix is resilient to changes in parameters, which can improve its robustness across different datasets and populations.	**Computational Complexity:** The use of SVM and HMM algorithms, especially for genome-wide analysis, may require significant computational resources and time. **Population Representativeness**: The accuracy of SupportMix depends on the representativeness of the parental populations used in the analysis, and biases or inaccuracies in the representation may affect the results.
6.	**ChromoPainter** [116]	**Utilization of Panel Data:** It utilizes information from large panel datasets that may be unrelated to the targeted admixed population, allowing for the reconstruction of ancestry patterns. **Worldwide Ancestry Reconstruction**: ChromoPainter can reconstruct both current and historical worldwide population ancestries, providing a broad perspective on genetic ancestry. **Accuracy with Few Reference Samples:** Simulation-based comparisons have shown that ChromoPainter has high accuracy at the genome-wide level, even when only a few reference samples are available. **Nonnegative Least Squares:** ChromoPainter utilizes Nonnegative Least Squares (NNLS) to deduce the painting information, which helps in inferring the ancestral components in the admixed population.	**Dependence on Panel Data:** ChromoPainter relies heavily on panel data, and the accuracy of the inference may be affected by the representativeness and quality of the panel datasets used. **Assumptions about Admixture:** Like any ancestry inference method, ChromoPainter relies on certain assumptions about the admixture process and the genetic similarity between populations, which may not always hold true in all cases.
7.	**RFMix** [118]	**Dependency Modeling:** The technique directly models the dependence of unseen factors (such as ancestries) on observable factors (such as alleles), allowing for a more accurate inference of local ancestry. **CRF Generated by Random Forest Models**: RFMix uses a Conditional Random Field (CRF) generated by random forest models learned on reference panels to estimate local ancestry within every window of each chromosome. **Improvement in Inference Accuracy**: After assigning ancestries to windows in admixed chromosomes, RFMix employs an expectation- maximization (EM) step to improve inference accuracy by better understanding haplotype trends in parental populations. **Precision and Speed:** Estimation of ancestry based on RFMix is reported to be more precise and faster compared to other techniques, such as LAMP (approximately 33 fold faster) and SupportMix (about 1.7 fold faster).	**Assumption of Known or Assumed Ancestry:** RFMix requires the ancestry of admixed individuals to be known or assumed, which may not always be the case in practical applications. **Population Representativeness**: The accuracy of RFMix depends on the representativeness of the reference panels used, and biases or inaccuracies in the representation may affect the results.
8.	**EILA** [115]	**Quantification of Ancestral Relatedness:** The method quantifies the relatedness of SNPs in admixed individuals to particular ancestral groups, providing a more nuanced understanding of local ancestry. **Breakpoint Determination**: EILA uses fused quantile regression to determine the breakpoints of ancestral haplotypes, which can improve the accuracy of ancestry inference. **Ancestry Inference at Each Site**: The k-means classifier is employed to infer ancestry at each site, allowing for detailed ancestry analysis at the individual SNP level. **Utilization of All Genotyped SNPs:** EILA relaxes the requirement of linkage equilibrium and uses all genotyped SNPs, rather than just unlinked loci, to enhance the power of inference.	**Computational Complexity:** EILA's three-phase approach, especially the use of fused quantile regression and k-means classifier, may introduce computational complexity, particularly for large datasets. **Accuracy and Sensitivity:** The accuracy of EILA may be sensitive to the choice of parameters and the assumptions made about the admixture process, which could impact the reliability of the inference.
9.	**ASPCA** [116]	**Tract Length Pattern Analysis**: ASPCA examines the tract length pattern of genomic regions related to different origins, which can help analyze biological models of modern demographic evolution.	**Model Assumptions**: The accuracy of ASPCA may depend on the assumptions made about the demographic models of modern human evolution, which may not always accurately reflect historical realities.
10.	**LOTER** [107]	**Wide Taxa Coverage:** LOTER is designed to infer local ancestry for a wide range of taxa, making it applicable to diverse species where biological parameters such as admixing timeframes and recombination rates are unknown. **Mimicking Approach:** LOTER utilizes the mimicking approach proposed by Li and Stephens (2003), which treats admixed individual haplotypes as a matrix of preexisting ancestral haplotypes from potential source parental populations. **Regularization Parameter:** It involves a smoothing control value called the regularization parameter (λ), which is dependent on a complex set of analytical and biological variables, including rates of mutation and recombination. LOTER averages solutions for various λ values to avoid the challenging selection of this parameter. **Phased Haplotypic Information**: LOTER requires phased haplotypic information for both reference and target populations and accounts for phase errors in the data. **Admixture Time Estimation:** The method estimates the time of admixture using restored ancestry sequences, providing insights into the timeframe since admixing occurred. **No Dependency on Genetic Maps:** LOTER does not require genetic maps, and there are no restrictions on the number of SNPs used, making it more flexible and applicable to a wide range	**Complex Parameter Selection:** The choice of the regularization parameter (λ) in LOTER is complex and involves a trade-off between penalizing switching among ancestral haplotypes and achieving accurate ancestry inference. This can make the method challenging to implement and interpret. **Accuracy in Admixture Time Estimation:** While LOTER provides estimates of the time of admixture, the accuracy of these estimates may depend on the specific parameters and assumptions used in the analysis. **Limited Applicability to Specific Scenarios:** LOTER may be more suitable for scenarios where biological parameters such as admixing timeframes and recombination rates are unknown or difficult to determine, limiting its applicability in other contexts where such parameters are well-defined.
11.	**FLARE** [123]	**Computational Efficiency:** FLARE incorporates computational methods originally developed for genotype imputation, allowing for remarkable computing performance even with large datasets containing tens of thousands of sequenced individuals. **Usage of Composite Reference Haplotypes:** FLARE utilizes composite reference haplotypes, which can speed up computation and improve accuracy in ancestry estimation. **Scalability:** FLARE can be utilized for datasets containing tens of thousands of sequenced individuals, indicating its scalability to large datasets.	**Dependency on Reference Haplotypes**: FLARE relies on composite reference haplotypes, which may need to be carefully constructed and curated to ensure accurate ancestry estimation. Biases or inaccuracies in the reference haplotypes could affect the results.
12.	**SALAI-Net** [125]	**Species Agnostic:** SALAI-Net is designed to be species-agnostic, which means it can be used for local ancestry inference in any species without the need for specific tuning or retraining. **Two-Stage Method:** SALAI-Net consists of two stages: a source comparing layer that provides initial estimations at the window level, followed by a smoother layer that improves these estimations by leveraging adjacent window data and reducing shortcomings. **Flexible Application:** Once SALAI-Net is trained on specific conditions, it can be applied to infer local ancestry across any species or group of ancestries without the need for further tuning or retraining, making it versatile and adaptable.	**Generalizability:** While SALAI-Net is designed to be flexible in its application, its performance may vary across different species or groups of ancestries, and its generalizability to all scenarios may not be guaranteed.
13.	**BCSYS** **(local ancestry classifier)** [126]	**Utilization of Large Reference Panels:** The use of large reference panels in BCSYS enables the calling of more breeds and increases accuracy by including more reference samples per breed. **Improved Accuracy for Mixed Breed Samples**: The BCSYS algorithm is specifically tuned to improve accuracy for samples with mixed-breed ancestry, enhancing its applicability to diverse populations. **Local Ancestry Classifier**: Unlike previous algorithms, BCSYS is a local ancestry classifier, meaning it not only calls the total proportion of breeds throughout an animal's genome but also assigns ancestry labels to specific small segments of chromosomes. **Machine Learning Model for Purebred Status Prediction**: BCSYS uses the local ancestry results to train a machine learning model that predicts the purebred status of an animal, providing additional insights into genetic ancestry. **Future Product Development:** The local ancestry classifier in BCSYS will drive future product development, detailing how an animal's physical traits relate to their individual ancestry, potentially leading to new applications in animal breeding and genetics.	**Model Complexity:** The use of machine learning models and the local ancestry classifier in BCSYS may introduce complexity, which could make it challenging to interpret and implement.
14.	**AFA (Ancestry-specific allele frequency)** [128]	**Estimation of Allele Frequencies:** AFA is designed to estimate the frequencies of biallelic variants in admixed populations with an unlimited number of ancestries, providing insights into the genetic composition of these populations. **Maximum-Likelihood Estimation:** AFA uses maximum-likelihood estimation by modeling the conditional probability of having an allele given proportions of genetic ancestries, allowing for a more accurate estimation of allele frequencies.	-

**Table 3:** Brief description of different ARG software that are commonly used in genetics.

**Software**	**Algorithm**	**Data Used**	**Inference**	**References**
*ARGweaver*	MCMC	54 Human genome sequence	Distinctive signs of natural selection are evident, such as regions with notably ancient ancestry linked to balancing selection and decreases in allele age at sites subject to directional selection	[156]
TARGet	TDA(Topological Data Analysis)	Genetic sequences of 112 Darwin’s finches	Lateral evolution observed in finches residing on the Galápagos Islands	[163, 164]
IRis	DSR(Dominant, Subdominant or Recombinant)	Genomic scale for hundreds of Human population data	Identified recombinations and local topological information	[154]
*ACG*	Bayesian MCMC	Ten sequences of length 10,000 sites under the standard neutral coalescent model	Identified the specific locations of individual recombination breakpoints not only across the sequence's length but also over time	[155]
KwARG	Heuristic-based parsimony	Binary matrix, or a multiple alignment in nucleotide or amino acid format	Discovered credible genealogical histories, often in the form of ancestral recombination graphs, characterized by minimal or near-minimal occurrences of posited recombination and mutation events	[163]
ARG-Needle	HMM	Genotype data of 337,464 UK Biobank individuals and to detect associations in 7 complex traits	Showed that utilizing large-scale inference of gene genealogies can be advantageous in the examination of complex traits	[162]
SARGE	Heuristic-based parsimony	279 modern human genomes, two high-coverage Neanderthal genomes, and one high-coverage Denisovan genome	Discovered that a mere 1.5 to 7% of the contemporary human genome is distinctive to humans, providing evidence of multiple episodes of adaptive changes specific to the modern human lineage	[151]

## LIST OF ABBREVIATIONS

AIMs: Ancestry-Informative Markers
ALD: Admixture Linkage Disequilibrium
ALL: Acute Lymphoblastic Leukemia
BGA: Biogeographic Ancestry
BLAD: Bovine Leukocyte Adhesion Deficiency
CGF: Continuous Gene Flow
CMD: Congenital Muscular Dystonia
CRF: Conditional Random Field
CVM: Complicated Vertebral Malformation
HI: Hybrid Isolation
HMM: Hidden Markov Model
I-BGA: Individual Biogeographical Ancestry
MAS: Marker-assisted Selection
MCI: Mild Cognitive Impairment
MCMC: Markov Chain Monte Carlo
MHs: Micro Haplotypes
MPS: Massively Parallel Sequencing
MSTN: Myostatin
PCA: Principal Component Analysis
QTL: Quantitative Trait Loci
SNP: Single Nucleotide Polymorphism
SSR: Simple Sequence Repeats
STRs: Short Tandem Repeats
SVM: Support Vector Machines

## References

[1] Tang H., Coram M., Wang P., Zhu X., Risch N. (2006). Reconstructing genetic ancestry blocks in admixed individuals.. Am. J. Hum. Genet..

[2] Popejoy A.B., Ritter D.I., Crooks K., Currey E., Fullerton S.M., Hindorff L.A., Koenig B., Ramos E.M., Sorokin E.P., Wand H., Wright M.W., Zou J., Gignoux C.R., Bonham V.L., Plon S.E., Bustamante C.D. (2018). The clinical imperative for inclusivity: Race, ethnicity, and ancestry (REA) in genomics.. Hum. Mutat..

[3] Long J.C. (1991). The genetic structure of admixed populations.. Genetics.

[4] Pfaff C.L., Parra E.J., Bonilla C., Hiester K., McKeigue P.M., Kamboh M.I., Hutchinson R.G., Ferrell R.E., Boerwinkle E., Shriver M.D. (2001). Population structure in admixed populations: Effect of admixture dynamics on the pattern of linkage disequilibrium.. Am. J. Hum. Genet..

[5] Facon B., Jarne P., Pointier J.P., David P. (2005). Hybridization and invasiveness in the freshwater snail *Melanoides tuberculata* : Hybrid vigour is more important than increase in genetic variance.. J. Evol. Biol..

[6] Martin A.R., Gignoux C.R., Walters R.K., Wojcik G.L., Neale B.M., Gravel S., Daly M.J., Bustamante C.D., Kenny E.E. (2017). Human demographic history impacts genetic risk prediction across diverse populations.. Am. J. Hum. Genet..

[7] Solkner J., Frkonja A., Raadsma H.W., Jonas E., Thaller G., Gootwine E., Seroussi C., Fuerst C., Danner E.C., Gredler B. (2010). Estimation of individual levels of admixture in crossbred populations from SNP chip data: Examples with sheep and cattle populations.. Interbull Bull..

[8] Anderson E.C. (2008). Bayesian inference of species hybrids using multilocus dominant genetic markers.. Philos. Trans. R. Soc. Lond. B Biol. Sci..

[9] Larmer S., Ventura R., Buzanskas M.E., Sargolzaei M., Schenkel F.S. (2014). Assessing admixture by quantifying breed composition to gain historical perspective on dairy cattle in Canada.. 10th World Congress on Genetics Applied to Livestock Production.

[10] Chakraborty R. (1986). Gene admixture in human populations: Models and predictions.. Am. J. Phys. Anthropol..

[11] Bryc K., Auton A., Nelson M.R., Oksenberg J.R., Hauser S.L., Williams S., Froment A., Bodo J.M., Wambebe C., Tishkoff S.A., Bustamante C.D. (2010). Genome-wide patterns of population structure and admixture in West Africans and African Americans.. Proc. Natl. Acad. Sci..

[12] Makina S.O., Muchadeyi F.C., van Köster M.E., MacNeil M.D., Maiwashe A. (2014). Genetic diversity and population structure among six cattle breeds in South Africa using a whole genome SNP panel.. Front. Genet..

[13] Khayatzadeh N., Mészáros G., Gredler B., Schnyder U., Curik I., Sölkner J. (2015). Prediction of global and local simmental and red holstein friesian admixture levels in swiss fleckvieh cattle.. Poljoprivreda.

[14] Kumar P., Freeman A.R., Loftus R.T., Gaillard C., Fuller D.Q., Bradley D.G. (2003). Admixture analysis of South Asian cattle.. Heredity.

[15] Dadi H., Tibbo M., Takahashi Y., Nomura K., Hanada H., Amano T. (2008). Microsatellite analysis reveals high genetic diversity but low genetic structure in Ethiopian indigenous cattle populations.. Anim. Genet..

[16] Schlötterer C., Tautz D. (1992). Slippage synthesis of simple sequence DNA.. Nucleic Acids Res..

[17] Innan H., Terauchi R., Miyashita N.T. (1997). Microsatellite polymorphism in natural populations of the wild plant Arabidopsis thaliana.. Genetics.

[18] McConnell R., Middlemist S., Scala C., Strassmann J.E., Queller D.C. (2007). An unusually low microsatellite mutation rate in Dictyostelium discoideum, an organism with unusually abundant microsatellites.. Genetics.

[19] Mukesh M., Sodhi M., Bhatia S. (2006). Microsatellite-based diversity analysis and genetic relationships of three Indian sheep breeds.. J. Anim. Breed. Genet..

[20] Hill E.W., Gu J., Eivers S.S., Fonseca R.G., McGivney B.A., Govindarajan P., Orr N., Katz L.M., MacHugh D. (2010). A sequence polymorphism in MSTN predicts sprinting ability and racing stamina in thoroughbred horses.. PLoS One.

[21] Charlier C., Coppieters W., Rollin F., Desmecht D., Agerholm J.S., Cambisano N., Carta E., Dardano S., Dive M., Fasquelle C., Frennet J.C., Hanset R., Hubin X., Jorgensen C., Karim L., Kent M., Harvey K., Pearce B.R., Simon P., Tama N., Nie H., Vandeputte S., Lien S., Longeri M., Fredholm M., Harvey R.J., Georges M. (2008). Highly effective SNP-based association mapping and management of recessive defects in livestock.. Nat. Genet..

[22] Sukhija N., Malik A.A., Devadasan J.M., Dash A., Bidyalaxmi K., Kumar R.D. (2023). Genome-wide selection signatures address trait specific candidate genes in cattle indigenous to arid regions of India.. Anim. Biotechnol..

[23] Goli R.C., Sukhija N., Rathi P., Chishi K.G., Koloi S., Malik A.A., Sree C C., Purohit P.B., Shetkar M., K K K. (2024). Unraveling the genetic tapestry of Indian chicken: A comprehensive study of molecular variations and diversity.. Ecol. Genet. Genom..

[24] Kanaka K.K., Sukhija N., Goli R.C., Singh S., Ganguly I., Dixit S.P., Dash A., Malik A.A. (2023). On the concepts and measures of diversity in the genomics era.. Curr. Plant Biol..

[25] Nievergelt C.M., Maihofer A.X., Shekhtman T., Libiger O., Wang X., Kidd K.K., Kidd J.R. (2013). Inference of human continental origin and admixture proportions using a highly discriminative ancestry informative 41-SNP panel.. Investig. Genet..

[26] Goddard M.E., Hayes B.J. (2007). Genomic selection.. J. Anim. Breed. Genet..

[27] Zhang K., Sun F. (2005). Assessing the power of tag SNPs in the mapping of quantitative trait loci (QTL) with extremal and random samples.. BMC Genet..

[28] Hayes B.J., Chamberlain A.J., McPARTLAN H., MacLeod I., Sethuraman L., Goddard M.E. (2007). Accuracy of marker-assisted selection with single markers and marker haplotypes in cattle.. Genet. Res..

[29] Eusebi P.G., Martinez A., Cortes O. (2019). Genomic tools for effective conservation of livestock breed diversity.. Diversity.

[30] Price A.L., Spencer C.C., Donnelly P. (2015). Progress and promise in understanding the genetic basis of common diseases.. Proc. Royal. Soc. B.

[31] Freeman A.R., Bradley D.G., Nagda S., Gibson J.P., Hanotte O. (2006). Combination of multiple microsatellite data sets to investigate genetic diversity and admixture of domestic cattle.. Anim. Genet..

[32] Behar D.M., Yunusbayev B., Metspalu M., Metspalu E., Rosset S., Parik J., Rootsi S., Chaubey G., Kutuev I., Yudkovsky G., Khusnutdinova E.K., Balanovsky O., Semino O., Pereira L., Comas D., Gurwitz D., Tamir B.B., Parfitt T., Hammer M.F., Skorecki K., Villems R. (2010). The genome-wide structure of the Jewish people.. Nature.

[33] Shi W., Ayub Q., Vermeulen M., Shao R., Zuniga S., van der Gaag K., de Knijff P., Kayser M., Xue Y., Tyler-Smith C. (2010). A worldwide survey of human male demographic history based on Y-SNP and Y-STR data from the HGDP-CEPH populations.. Mol. Biol. Evol..

[34] Frkonja A., Gredler B., Schnyder U., Curik I., Sölkner J. (2012). Prediction of breed composition in an admixed cattle population.. Anim. Genet..

[35] Lenstra J.A., Groeneveld L.F., Eding H., Kantanen J., Williams J.L., Taberlet P., Nicolazzi E.L., Sölkner J., Simianer H., Ciani E., Garcia J.F., Bruford M.W., Ajmone-Marsan P., Weigend S. (2012). Molecular tools and analytical approaches for the characterization of farm animal genetic diversity.. Anim. Genet..

[36] McKay S.D., Schnabel R.D., Murdoch B.M., Matukumalli L.K., Aerts J., Coppieters W., Crews D., Neto E.D., Gill C.A., Gao C., Mannen H., Wang Z., Van Tassell C.P., Williams J.L., Taylor J.F., Moore S.S. (2008). An assessment of population structure in eight breeds of cattle using a whole genome SNP panel.. BMC Genet..

[37] Dawson E. (1999). SNP maps: More markers needed?. Mol. Med. Today.

[38] Vignal A., Milan D., SanCristobal M., Eggen A. (2002). A review on SNP and other types of molecular markers and their use in animal genetics.. Genet. Sel. Evol..

[39] Hong E.P., Park J.W. (2012). Sample size and statistical power calculation in genetic association studies.. Genomics Inform..

[40] Prasad A., Schnabel R.D., McKay S.D., Murdoch B., Stothard P., Kolbehdari D., Wang Z., Taylor J.F., Moore S.S. (2008). Linkage disequilibrium and signatures of selection on chromosomes 19 and 29 in beef and dairy cattle.. Anim. Genet..

[41] de Roos A.P.W., Hayes B.J., Spelman R.J., Goddard M.E. (2008). Linkage disequilibrium and persistence of phase in Holstein-Friesian, Jersey and Angus cattle.. Genetics.

[42] Ishii A., Yamaji K., Uemoto Y., Sasago N., Kobayashi E., Kobayashi N., Matsuhashi T., Maruyama S., Matsumoto H., Sasazaki S., Mannen H. (2013). Genome-wide association study for fatty acid composition in J apanese B lack cattle.. Anim. Sci. J..

[43] Gunia M., Saintilan R., Venot E., Hozé C., Fouilloux M.N., Phocas F. (2014). Genomic prediction in French charolais beef cattle using high-density single nucleotide polymorphism markers1.. J. Anim. Sci..

[44] Elsik C.G., Tellam R.L., Worley K.C., Gibbs R.A., Muzny D.M., Weinstock G.M., Adelson D.L., Eichler E.E., Elnitski L., Guigó R., Hamernik D.L., Kappes S.M., Lewin H.A., Lynn D.J., Nicholas F.W., Reymond A., Rijnkels M., Skow L.C., Zdobnov E.M., Schook L., Womack J., Alioto T., Antonarakis S.E., Astashyn A., Chapple C.E., Chen H.C., Chrast J., Câmara F., Ermolaeva O., Henrichsen C.N., Hlavina W., Kapustin Y., Kiryutin B., Kitts P., Kokocinski F., Landrum M., Maglott D., Pruitt K., Sapojnikov V., Searle S.M., Solovyev V., Souvorov A., Ucla C., Wyss C., Anzola J.M., Gerlach D., Elhaik E., Graur D., Reese J.T., Edgar R.C., McEwan J.C., Payne G.M., Raison J.M., Junier T., Kriventseva E.V., Eyras E., Plass M., Donthu R., Larkin D.M., Reecy J., Yang M.Q., Chen L., Cheng Z., Chitko-McKown C.G., Liu G.E., Matukumalli L.K., Song J., Zhu B., Bradley D.G., Brinkman F.S.L., Lau L.P.L., Whiteside M.D., Walker A., Wheeler T.T., Casey T., German J.B., Lemay D.G., Maqbool N.J., Molenaar A.J., Seo S., Stothard P., Baldwin C.L., Baxter R., Brinkmeyer-Langford C.L., Brown W.C., Childers C.P., Connelley T., Ellis S.A., Fritz K., Glass E.J., Herzig C.T.A., Iivanainen A., Lahmers K.K., Bennett A.K., Dickens C.M., Gilbert J.G.R., Hagen D.E., Salih H., Aerts J., Caetano A.R., Dalrymple B., Garcia J.F., Gill C.A., Hiendleder S.G., Memili E., Spurlock D., Williams J.L., Alexander L., Brownstein M.J., Guan L., Holt R.A., Jones S.J.M., Marra M.A., Moore R., Moore S.S., Roberts A., Taniguchi M., Waterman R.C., Chacko J., Chandrabose M.M., Cree A., Dao M.D., Dinh H.H., Gabisi R.A., Hines S., Hume J., Jhangiani S.N., Joshi V., Kovar C.L., Lewis L.R., Liu Y., Lopez J., Morgan M.B., Nguyen N.B., Okwuonu G.O., Ruiz S.J., Santibanez J., Wright R.A., Buhay C., Ding Y., Dugan-Rocha S., Herdandez J., Holder M., Sabo A., Egan A., Goodell J., Wilczek-Boney K., Fowler G.R., Hitchens M.E., Lozado R.J., Moen C., Steffen D., Warren J.T., Zhang J., Chiu R., Schein J.E., Durbin K.J., Havlak P., Jiang H., Liu Y., Qin X., Ren Y., Shen Y., Song H., Bell S.N., Davis C., Johnson A.J., Lee S., Nazareth L.V., Patel B.M., Pu L.L., Vattathil S., Williams R.L., Curry S., Hamilton C., Sodergren E., Wheeler D.A., Barris W., Bennett G.L., Eggen A., Green R.D., Harhay G.P., Hobbs M., Jann O., Keele J.W., Kent M.P., Lien S., McKay S.D., McWilliam S., Ratnakumar A., Schnabel R.D., Smith T., Snelling W.M., Sonstegard T.S., Stone R.T., Sugimoto Y., Takasuga A., Taylor J.F., Van Tassell C.P., MacNeil M.D., Abatepaulo A.R.R., Abbey C.A., Ahola V., Almeida I.G., Amadio A.F., Anatriello E., Bahadue S.M., Biase F.H., Boldt C.R., Carroll J.A., Carvalho W.A., Cervelatti E.P., Chacko E., Chapin J.E., Cheng Y., Choi J., Colley A.J., de Campos T.A., De Donato M., Santos I.K.F.M., de Oliveira C.J.F., Deobald H., Devinoy E., Donohue K.E., Dovc P., Eberlein A., Fitzsimmons C.J., Franzin A.M., Garcia G.R., Genini S., Gladney C.J., Grant J.R., Greaser M.L., Green J.A., Hadsell D.L., Hakimov H.A., Halgren R., Harrow J.L., Hart E.A., Hastings N., Hernandez M., Hu Z.L., Ingham A., Iso-Touru T., Jamis C., Jensen K., Kapetis D., Kerr T., Khalil S.S., Khatib H., Kolbehdari D., Kumar C.G., Kumar D., Leach R., Lee J.C.M., Li C., Logan K.M., Malinverni R., Marques E., Martin W.F., Martins N.F., Maruyama S.R., Mazza R., McLean K.L., Medrano J.F., Moreno B.T., Moré D.D., Muntean C.T., Nandakumar H.P., Nogueira M.F.G., Olsaker I., Pant S.D., Panzitta F., Pastor R.C.P., Poli M.A., Poslusny N., Rachagani S., Ranganathan S., Razpet A., Riggs P.K., Rincon G., Osorio R.N., Zas R.S.L., Romero N.E., Rosenwald A., Sando L., Schmutz S.M., Shen L., Sherman L., Southey B.R., Lutzow Y.S., Sweedler J.V., Tammen I., Telugu B.P.V.L., Urbanski J.M., Utsunomiya Y.T., Verschoor C.P., Waardenberg A.J., Wang Z., Ward R., Weikard R., Welsh T.H., White S.N., Wilming L.G., Wunderlich K.R., Yang J., Zhao F.Q. (2009). The genome sequence of taurine cattle: A window to ruminant biology and evolution.. Science.

[45] Gibbs R.A., Taylor J.F., Van Tassell C.P., Barendse W., Eversole K.A., Gill C.A., Green R.D., Hamernik D.L., Kappes S.M., Lien S., Matukumalli L.K., McEwan J.C., Nazareth L.V., Schnabel R.D., Weinstock G.M., Wheeler D.A., Ajmone-Marsan P., Boettcher P.J., Caetano A.R., Garcia J.F., Hanotte O., Mariani P., Skow L.C., Sonstegard T.S., Williams J.L., Diallo B., Hailemariam L., Martinez M.L., Morris C.A., Silva L.O.C., Spelman R.J., Mulatu W., Zhao K., Abbey C.A., Agaba M., Araujo F.R., Bunch R.J., Burton J., Gorni C., Olivier H., Harrison B.E., Luff B., Machado M.A., Mwakaya J., Plastow G., Sim W., Smith T., Thomas M.B., Valentini A., Williams P., Womack J., Woolliams J.A., Liu Y., Qin X., Worley K.C., Gao C., Jiang H., Moore S.S., Ren Y., Song X.Z., Bustamante C.D., Hernandez R.D., Muzny D.M., Patil S., San Lucas A., Fu Q., Kent M.P., Vega R., Matukumalli A., McWilliam S., Sclep G., Bryc K., Choi J., Gao H., Grefenstette J.J., Murdoch B., Stella A., Villa-Angulo R., Wright M., Aerts J., Jann O., Negrini R., Goddard M.E., Hayes B.J., Bradley D.G., Barbosa da Silva M., Lau L.P.L., Liu G.E., Lynn D.J., Panzitta F., Dodds K.G. (2009). Genome-wide survey of SNP variation uncovers the genetic structure of cattle breeds.. Science.

[46] Phillips C., Salas A., Sánchez J.J., Fondevila M., Tato G.A., Dios A.J., Calaza M., de Cal M.C., Ballard D., Lareu M.V., Carracedo Á. (2007). Inferring ancestral origin using a single multiplex assay of ancestry-informative marker SNPs.. Forensic Sci. Int. Genet..

[47] Halder I., Shriver M., Thomas M., Fernandez J.R., Frudakis T. (2008). A panel of ancestry informative markers for estimating individual biogeographical ancestry and admixture from four continents: Utility and applications.. Hum. Mutat..

[48] Morin P.A., Luikart G., Wayne R.K. (2004). SNPs in ecology, evolution and conservation.. Trends Ecol. Evol..

[49] Lewis J., Abas Z., Dadousis C., Lykidis D., Paschou P., Drineas P. (2011). Tracing cattle breeds with principal components analysis ancestry informative SNPs.. PLoS One.

[50] Winkler C.A., Nelson G.W., Smith M.W. (2010). Admixture mapping comes of age.. Annu. Rev. Genomics Hum. Genet..

[51] Banks M.A., Eichert W., Olsen J.B. (2003). Which genetic loci have greater population assignment power?. Bioinformatics.

[52] Bromaghin J.F. (2008). bels : Backward elimination locus selection for studies of mixture composition or individual assignment.. Mol. Ecol. Resour..

[53] Helyar S.J., Hemmer-Hansen J., Bekkevold D., Taylor M.I., Ogden R., Limborg M.T., Cariani A., Maes G.E., Diopere E., Carvalho G.R., Nielsen E.E. (2011). Application of SNPs for population genetics of nonmodel organisms: new opportunities and challenges.. Mol. Ecol. Resour..

[54] Rosenberg N.A., Li L.M., Ward R., Pritchard J.K. (2003). Informativeness of genetic markers for inference of ancestry.. Am. J. Hum. Genet..

[55] Shannon C.E. (1948). A mathematical theory of communication.. Bell Syst. Tech. J..

[56] Shriver M.D., Smith M.W., Jin L., Marcini A., Akey J.M., Deka R., Ferrell R.E. (1997). Ethnic-affiliation estimation by use of population-specific DNA markers.. Am. J. Hum. Genet..

[57] Wright S. (1951). The genetical structure of populations.. Ann. Eugen..

[58] Weir B.S., Cockerham C.C. (1984). Estimating F-statistics for the analysis of population structure.. Evolution.

[59] Kavakiotis I., Samaras P., Triantafyllidis A., Vlahavas I. (2017). FIFS: A data mining method for informative marker selection in high dimensional population genomic data.. Comput. Biol. Med..

[60] Shriner D. (2013). Overview of admixture mapping.. Curr. Protoc. Hum. Genet..

[61] Padhukasahasram B. (2014). Inferring ancestry from population genomic data and its applications.. Front. Genet..

[62] Falush D., Stephens M., Pritchard J.K. (2003). Inference of population structure using multilocus genotype data: Linked loci and correlated allele frequencies.. Genetics.

[63] Pritchard J.K., Stephens M., Donnelly P. (2000). Inference of population structure using multilocus genotype data.. Genetics.

[64] Alexander D.H., Novembre J., Lange K. (2009). Fast model-based estimation of ancestry in unrelated individuals.. Genome Res..

[65] Liu Y., Nyunoya T., Leng S., Belinsky S.A., Tesfaigzi Y., Bruse S. (2013). Softwares and methods for estimating genetic ancestry in human populations.. Hum. Genomics.

[66] Skotte L., Korneliussen T.S., Albrechtsen A. (2013). Estimating individual admixture proportions from next generation sequencing data.. Genetics.

[67] Bertorelle G., Excoffier L. (1998). Inferring admixture proportions from molecular data.. Mol. Biol. Evol..

[68] Rosenberg N.A., Pritchard J.K., Weber J.L., Cann H.M., Kidd K.K., Zhivotovsky L.A., Feldman M.W. (2002). Genetic structure of human populations.. Science.

[69] Edea Z., Dadi H., Kim S.W., Dessie T., Lee T., Kim H., Kim J.J., Kim K.S. (2013). Genetic diversity, population structure and relationships in indigenous cattle populations of Ethiopia and Korean Hanwoo breeds using SNP markers.. Front. Genet..

[70] Patterson N., Price A.L., Reich D. (2006). Population structure and eigenanalysis.. PLoS Genet..

[71] Gao X., Starmer J. (2007). Human population structure detection *via* multilocus genotype clustering.. BMC Genet..

[72] Menozzi P., Piazza A., Cavalli-Sforza L. (1978). Synthetic maps of human gene frequencies in Europeans.. Science.

[73] Bouaziz M., Ambroise C., Guedj M. (2011). Accounting for population stratification in practice: A comparison of the main strategies dedicated to genome-wide association studies.. PLoS One.

[74] Siegel S. (1957). Nonparametric Statistics.. Am. Stat..

[75] Beasley T.M., Erickson S., Allison D.B. (2009). Rank-based inverse normal transformations are increasingly used, but are they merited?. Behav. Genet..

[76] Girma M., Banerjee S., Birhanu T. (2020). Breeding practice and phenotypic characteristics of indigenous Woyito-Guji goat breeds reared in Nyangatom and Malle pastoral and agro-pastoral districts of SNNPR, Ethiopia.. Int. J. Animal Sci..

[77] Potvin C., Roff D.A. (1993). Distribution-free and robust statistical methods: viable alternatives to parametric statistics.. Ecology.

[78] Gianola D., Fernando R.L., Stella A. (2006). Genomic-assisted prediction of genetic value with semiparametric procedures.. Genetics.

[79] Nordborg M., Tavaré S. (2002). Linkage disequilibrium: What history has to tell us.. Trends Genet..

[80] Vila M., Romaní V.J.R., Björklund M. (2005). The importance of time scale and multiple refugia: Incipient speciation and admixture of lineages in the butterfly Erebia triaria (Nymphalidae).. Mol. Phylogenet. Evol..

[81] Moorjani P., Patterson N., Hirschhorn J.N., Keinan A., Hao L., Atzmon G., Burns E., Ostrer H., Price A.L., Reich D. (2011). The history of African gene flow into Southern Europeans, Levantines, and Jews.. PLoS Genet..

[82] Pugach I., Matveyev R., Wollstein A., Kayser M., Stoneking M. (2011). Dating the age of admixture *via* wavelet transform analysis of genome-wide data.. Genome Biol..

[83] Sankararaman S., Patterson N., Li H., Pääbo S., Reich D. (2012). The date of interbreeding between Neandertals and modern humans.. PLoS Genet.

[84] Loh P.R., Lipson M., Patterson N., Moorjani P., Pickrell J.K., Reich D., Berger B. (2013). Inferring admixture histories of human populations using linkage disequilibrium.. Genetics.

[85] McTavish E.J., Hillis D.M. (2014). A genomic approach for distinguishing between recent and ancient admixture as applied to cattle.. J. Hered..

[86] Hellenthal G., Busby G.B., Band G., Wilson J.F., Capelli C., Falush D., Myers S. (2014). A genetic atlas of human admixture history.. science.

[87] Avadhanam S., Williams A.L. (2022). Simultaneous inference of parental admixture proportions and admixture times from unphased local ancestry calls.. Am. J. Hum. Genet..

[88] Chakraborty R., Weiss K.M. (1988). Admixture as a tool for finding linked genes and detecting that difference from allelic association between loci.. Proc. Natl. Acad. Sci..

[89] McKeigue P.M. (1998). Mapping genes that underlie ethnic differences in disease risk: methods for detecting linkage in admixed populations, by conditioning on parental admixture.. Am. J. Hum. Genet..

[90] Hoggart C.J., Shriver M.D., Kittles R.A., Clayton D.G., McKeigue P.M. (2004). Design and analysis of admixture mapping studies.. Am. J. Hum. Genet..

[91] Zhang C., Chen K., Seldin M.F., Li H. (2004). A hidden Markov modeling approach for admixture mapping based on case-control data.. Genet. Epidemiol..

[92] Zhu X., Zhang S., Tang H., Cooper R. (2006). A classical likelihood based approach for admixture mapping using EM algorithm.. Hum. Genet..

[93] Patterson N., Hattangadi N., Lane B., Lohmueller K.E., Hafler D.A., Oksenberg J.R., Hauser S.L., Smith M.W., O’Brien S.J., Altshuler D., Daly M.J., Reich D. (2004). Methods for high-density admixture mapping of disease genes.. Am. J. Hum. Genet..

[94] Hoggart C.J., Parra E.J., Shriver M.D., Bonilla C., Kittles R.A., Clayton D.G., McKeigue P.M. (2003). Control of confounding of genetic associations in stratified populations.. Am. J. Hum. Genet..

[95] Tang H., Choudhry S., Mei R., Morgan M., Rodriguez-Cintron W., Burchard E.G., Risch N.J. (2007). Recent genetic selection in the ancestral admixture of Puerto Ricans.. Am. J. Hum. Genet..

[96] Jin W., Xu S., Wang H., Yu Y., Shen Y., Wu B., Jin L. (2012). Genome-wide detection of natural selection in African Americans pre- and post-admixture.. Genome Res..

[97] Jones O.R., Wang J. (2012). A comparison of four methods for detecting weak genetic structure from marker data.. Ecol. Evol..

[98] Bertorelle G., Raffini F., Bosse M., Bortoluzzi C., Iannucci A., Trucchi E., Morales H.E., van Oosterhout C. (2022). Genetic load: genomic estimates and applications in non-model animals.. Nat. Rev. Genet..

[99] Oleksyk T.K., Smith M.W., O’Brien S.J. (2010). Genome-wide scans for footprints of natural selection.. Philos. Trans. R. Soc. Lond. B Biol. Sci..

[100] Payseur B.A., Rieseberg L.H. (2016). A genomic perspective on hybridization and speciation.. Mol. Ecol..

[101] Yougbaré B., Ouédraogo D., Tapsoba A.S.R., Soudré A., Zoma B.L., terWengel O.P., Moumouni S., Koné O.S., Wurzinger M., Tamboura H.H., Traoré A., Mwai O.A., Sölkner J., Khayatzadeh N., Mészáros G., Burger P.A. (2021). Local ancestry to identify selection in response to trypanosome infection in Baoulé x zebu crossbred cattle in Burkina Faso.. Front. Genet..

[102] Gautier M., Naves M. (2011). Footprints of selection in the ancestral admixture of a New World Creole cattle breed.. Mol. Ecol..

[103] Detig C.R., Nielsen R. (2017). A hidden Markov model approach for simultaneously estimating local ancestry and admixture time using next generation sequence data in samples of arbitrary ploidy.. PLoS Genet..

[104] Sankararaman S., Sridhar S., Kimmel G., Halperin E. (2008). Estimating local ancestry in admixed populations.. Am. J. Hum. Genet..

[105] Baran Y., Pasaniuc B., Sankararaman S., Torgerson D.G., Gignoux C., Eng C., Cintron R.W., Chapela R., Ford J.G., Avila P.C., Santana R.J., Burchard E.G., Halperin E. (2012). Fast and accurate inference of local ancestry in Latino populations.. Bioinformatics.

[106] Paşaniuc B., Sankararaman S., Kimmel G., Halperin E. (2009). Inference of locus-specific ancestry in closely related populations.. Bioinformatics.

[107] Li N., Stephens M. (2003). Modeling linkage disequilibrium and identifying recombination hotspots using single-nucleotide polymorphism data.. Genetics.

[108] Yang J.J., Cheng C., Devidas M., Cao X., Fan Y., Campana D., Yang W., Neale G., Cox N.J., Scheet P., Borowitz M.J., Winick N.J., Martin P.L., Willman C.L., Bowman W.P., Camitta B.M., Carroll A., Reaman G.H., Carroll W.L., Loh M., Hunger S.P., Pui C.H., Evans W.E., Relling M.V. (2011). Ancestry and pharmacogenomics of relapse in acute lymphoblastic leukemia.. Nat. Genet..

[109] Brisbin A., Bryc K., Byrnes J., Zakharia F., Omberg L., Degenhardt J., Reynolds A., Ostrer H., Mezey J.G., Bustamante C.D. (2012). PCAdmix: Principal components-based assignment of ancestry along each chromosome in individuals with admixed ancestry from two or more populations.. Hum. Biol..

[110] Omberg L., Salit J., Hackett N., Fuller J., Matthew R., Chouchane L., Rodriguez-Flores J.L., Bustamante C., Crystal R.G., Mezey J.G. (2012). Inferring genome-wide patterns of admixture in Qataris using fifty-five ancestral populations.. BMC Genet..

[111] Mendizabal I., Lao O., Marigorta U.M., Wollstein A., Gusmão L., Ferak V., Ioana M., Jordanova A., Kaneva R., Kouvatsi A., Kučinskas V., Makukh H., Metspalu A., Netea M.G., de Pablo R., Pamjav H., Radojkovic D., Rolleston S.J.H., Sertic J., Macek M., Comas D., Kayser M. (2012). Reconstructing the population history of European Romani from genome-wide data.. Curr. Biol..

[112] Lawson D.J., Hellenthal G., Myers S., Falush D. (2012). Inference of population structure using dense haplotype data.. PLoS Genet..

[113] Spangenberg L., Fariello M.I., Arce D., Illanes G., Greif G., Shin J.Y., Yoo S.K., Seo J.S., Robello C., Kim C., Novembre J., Sans M., Naya H. (2021). Indigenous ancestry and admixture in the Uruguayan population.. Front. Genet..

[114] Maples B.K., Gravel S., Kenny E.E., Bustamante C.D. (2013). RFMix: A discriminative modeling approach for rapid and robust local-ancestry inference.. Am. J. Hum. Genet..

[115] Yang J. J., Li J., Buu A., Williams L. K., Yang M. J. J. (2013). Efficient inference of local ancestry.. Bioinformatics.

[116] Moreno-Estrada A., Gravel S., Zakharia F., McCauley J.L., Byrnes J.K., Gignoux C.R., Tello O.P.A., Martínez R.J., Hedges D.J., Morris R.W., Eng C., Sandoval K., Acevedo A.S., Norman P.J., Layrisse Z., Parham P., Martínez-Cruzado J.C., Burchard E.G., Cuccaro M.L., Martin E.R., Bustamante C.D. (2013). Reconstructing the population genetic history of the Caribbean.. PLoS Genet..

[117] Kumar L., Farias K., Prakash S., Mishra A., Mustak M.S., Rai N., Thangaraj K. (2021). Dissecting the genetic history of the roman catholic populations of West Coast India.. Hum. Genet..

[118] Dias-Alves T., Mairal J., Blum M.G.B. (2018). Loter: A software package to infer local ancestry for a wide range of species.. Mol. Biol. Evol..

[119] Daya M., van der Merwe L., Gignoux C.R., van Helden P.D., Möller M., Hoal E.G. (2014). Using multi-way admixture mapping to elucidate TB susceptibility in the South African Coloured population.. BMC Genomics.

[120] Wu M.Y., Forcina G., Low G.W., Sadanandan K.R., Gwee C.Y., van Grouw H., Wu S., Edwards S.V., Baldwin M.W., Rheindt F.E. (2023). Historic samples reveal loss of wild genotype through domestic chicken introgression during the Anthropocene.. PLoS Genet..

[121] Lucas-Sánchez M., Fadhlaoui-Zid K., Comas D. (2023). The genomic analysis of current-day North African populations reveals the existence of trans-Saharan migrations with different origins and dates.. Hum. Genet..

[122] Wedger M.J., Roma-Burgos N., Olsen K.M. (2022). Genomic revolution of US weedy rice in response to 21st century agricultural technologies.. Commun. Biol..

[123] Browning S.R., Waples R.K., Browning B.L. (2023). Fast, accurate local ancestry inference with FLARE.. Am. J. Hum. Genet..

[124] Lawrence E.S., Gu W., Bohlender R.J., Ramirez A.C., Cole A.M., Yu J.J., Hu H., Heinrich E.C., O’Brien K.A., Vasquez C.A., Cowan Q.T., Bruck P.T., Mercader K., Alotaibi M., Long T., Hall J.E., Moya E.A., Bauk M.A., Reeves J.J., Kong M.C., Salem R.M., Vizcardo-Galindo G., Macarlupu J.L., Mujíca F.R., Bermudez D., Corante N., Gaio E., Fox K.P., Salomaa V., Havulinna A.S., Murray A.J., Malhotra A., Powel F.L., Jain M., Komor A.C., Cavalleri G.L., Huff C.D., Villafuerte F.C., Simonson T.S. (2024). Functional *EPAS1*/*HIF2A* missense variant is associated with hematocrit in Andean highlanders.. Sci. Adv..

[125] Sabat O.B., Montserrat M.D., Nieto G.X., Ioannidis A.G. (2022). SALAI-Net: Species-agnostic local ancestry inference network.. Bioinformatics.

[126] Garrigan D., Huff J., Foran C.R. (2023). BCSYS: An accurate and scalable local ancestry classifier.. https://www.wisdompanel.com/downloads/wp-breed-detection.pdf.

[127] Freyer J., Labadie J.D., Huff J.T., Denyer M., Forman O.P., Foran C.R., Donner J. (2024). Association of *FGF4L1* retrogene insertion with prolapsed gland of the nictitans (Cherry Eye) in dogs.. Genes.

[128] Hershkovitz G.E., Xia R., Yang Y., Spitzer B., Tarraf W., Vásquez P.M., Lipton R.B., Daviglus M., Argos M., Cai J., Kaplan R., Fornage M., DeCarli C., Gonzalez H.M., Sofer T. (2023). Interaction analysis of ancestry-enriched variants with APOE-ɛ4 on MCI in the Study of Latinos-Investigation of Neurocognitive Aging.. Sci. Rep..

[129] Quillen E.E., Bauchet M., Bigham A.W., Burbano D.M.E., Faust F.X., Klimentidis Y.C., Mao X., Stoneking M., Shriver M.D. (2012). OPRM1 and EGFR contribute to skin pigmentation differences between Indigenous Americans and Europeans.. Hum. Genet..

[130] Cerqueira C.C.S., Paixão-Côrtes V.R., Zambra F.M.B., Salzano F.M., Hünemeier T., Bortolini M.C. (2012). Predicting *homo* pigmentation phenotype through genomic data: From neanderthal to James Watson.. Am. J. Hum. Biol..

[131] Gerstenblith M.R., Shi J., Landi M.T. (2010). Genome-wide association studies of pigmentation and skin cancer: A review and meta-analysis.. Pigment Cell Melanoma Res..

[132] Sturm R.A., Duffy D.L. (2012). Human pigmentation genes under environmental selection.. Genome Biol..

[133] Sukhija N., Kanaka K.K., Goli R.C., Kapoor P., Sivalingam J., Verma A., Sharma R., Tripathi S.B., Malik A.A. (2023). The flight of chicken genomics and allied omics-a mini review.. Ecol. Genet. Genom..

[134] Kopp J.B., Smith M.W., Nelson G.W., Johnson R.C., Freedman B.I., Bowden D.W., Oleksyk T., McKenzie L.M., Kajiyama H., Ahuja T.S., Berns J.S., Briggs W., Cho M.E., Dart R.A., Kimmel P.L., Korbet S.M., Michel D.M., Mokrzycki M.H., Schelling J.R., Simon E., Trachtman H., Vlahov D., Winkler C.A. (2008). MYH9 is a major-effect risk gene for focal segmental glomerulosclerosis.. Nat. Genet..

[135] Norton H.L., Kittles R.A., Parra E., McKeigue P., Mao X., Cheng K., Canfield V.A., Bradley D.G., McEvoy B., Shriver M.D. (2006). Genetic evidence for the convergent evolution of light skin in Europeans and East Asians.. Mol. Biol. Evol..

[136] Beleza S., Johnson N.A., Candille S.I., Absher D.M., Coram M.A., Lopes J., Campos J., Araújo I.I., Anderson T.M., Vilhjálmsson B.J., Nordborg M., Correia e Silva A., Shriver M.D., Rocha J., Barsh G.S., Tang H., Tang H. (2013). Genetic architecture of skin and eye color in an African-European admixed population.. PLoS Genet..

[137] Pickrell J.K., Reich D. (2014). Toward a new history and geography of human genes informed by ancient DNA.. Trends Genet..

[138] Harding R.M., Tomlinson J.B., Ray A.J., Wakamatsu K., Rees J.L., McKenzie C.A. (2003). Phenotypic expression of melanocortin-1 receptor mutations in Black Jamaicans.. J. Invest. Dermatol..

[139] Chaitanya L., Ralf A., Oven M., Kupiec T., Chang J., Lagacé R., Kayser M. (2015). Simultaneous whole mitochondrial genome sequencing with short overlapping amplicons suitable for degraded DNA using the ion torrent personal genome machine.. Hum. Mutat..

[140] Ralf A., van Oven M., González M.D., de Knijff P., van der Beek K., Wootton S., Lagacé R., Kayser M. (2019). Forensic Y-SNP analysis beyond SNaPshot: High-resolution Y-chromosomal haplogrouping from low quality and quantity DNA using Ion AmpliSeq and targeted massively parallel sequencing.. Forensic Sci. Int. Genet..

[141] Phillips C. (2015). Forensic genetic analysis of bio-geographical ancestry.. Forensic Sci. Int. Genet..

[142] Phillips C., Devesse L., Ballard D., van Weert L., de la Puente M., Melis S., Iglesias A.V., Aradas F.A., Oldroyd N., Holt C., Court S.D., Carracedo Á., Lareu M.V. (2018). Global patterns of STR sequence variation: Sequencing the CEPH human genome diversity panel for 58 forensic STRs using the Illumina ForenSeq DNA Signature Prep Kit.. Electrophoresis.

[143] Pitt D., Bruford M.W., Barbato M., terWengel O.P., Martínez R., Sevane N. (2019). Demography and rapid local adaptation shape Creole cattle genome diversity in the tropics.. Evol. Appl..

[144] Noyes H., Brass A., Obara I., Anderson S., Archibald A.L., Bradley D.G., Fisher P., Freeman A., Gibson J., Gicheru M., Hall L., Hanotte O., Hulme H., McKeever D., Murray C., Oh S.J., Tate C., Smith K., Tapio M., Wambugu J., Williams D.J., Agaba M., Kemp S.J. (2011). Genetic and expression analysis of cattle identifies candidate genes in pathways responding to *Trypanosoma congolense* infection.. Proc. Natl. Acad. Sci..

[145] Ward J.A., McHugo G.P., Dover M.J., Hall T.J., Ng’ang’a S.I., Sonstegard T.S., Bradley D.G., Frantz L.A.F., Townshend S.M., MacHugh D.E. (2022). Genome-wide local ancestry and evidence for mitonuclear coadaptation in African hybrid cattle populations.. iScience.

[146] Griffiths R.C., Marjoram P., Donnelly P., Tavare S. (1997). An ancestral recombination graph.. Progress in Population Genetics and Human Evolution..

[147] Rasmussen M.D., Hubisz M.J., Gronau I., Siepel A. (2014). Genome-wide inference of ancestral recombination graphs.. PLoS Genet..

[148] Martin D.P., Lemey P., Posada D. (2011). Analysing recombination in nucleotide sequences.. Mol. Ecol. Resour..

[149] Hubisz M., Siepel A. (2020). Inference of ancestral recombination graphs using ARGweaver.. Methods Mol Biol.

[150] Marjoram P., Wall J.D. (2006). Fast “coalescent” simulation.. BMC Genet..

[151] Schaefer N.K., Shapiro B., Green R.E. (2021). An ancestral recombination graph of human, Neanderthal, and Denisovan genomes.. Sci. Adv..

[152] Buendia P., Narasimhan G. (2006). Serial NetEvolve: A flexible utility for generating serially-sampled sequences along a tree or recombinant network.. Bioinformatics.

[153] McGill J.R., Walkup E.A., Kuhner M.K. (2013). GraphML specializations to codify ancestral recombinant graphs.. Front. Genet..

[154] Javed A., Pybus M., Melé M., Utro F., Bertranpetit J., Calafell F., Parida L. (2011). IRiS: Construction of ARG networks at genomic scales.. Bioinformatics.

[155] O’Fallon B.D. (2013). ACG: Rapid inference of population history from recombining nucleotide sequences.. BMC Bioinformatics.

[156] Rasmussen M. D., Siepel A. (2013). Genome-wide inference of ancestral recombination graphs.. arXiv1306.5110v2.

[157] Mirzaei S., Wu Y. (2017). RENT+: An improved method for inferring local genealogical trees from haplotypes with recombination.. Bioinformatics.

[158] Duchemin W., Anselmetti Y., Patterson M., Ponty Y., Bérard S., Chauve C., Scornavacca C., Daubin V., Tannier E. (2017). DeCoSTAR: Reconstructing the ancestral organization of genes or genomes using reconciled phylogenies.. Genome Biol. Evol..

[159] Speidel L., Forest M., Shi S., Myers S.R. (2019). A method for genome-wide genealogy estimation for thousands of samples.. Nat. Genet..

[160] Zhang B.C., Biddanda A., Palamara P.F. (2021). Biobank-scale inference of ancestral recombination graphs enables genealogy-based mixed model association of complex traits.. bioRxiv.

[161] Ignatieva A., Lyngsø R.B., Jenkins P.A., Hein J. (2021). KwARG: Parsimonious reconstruction of ancestral recombination graphs with recurrent mutation.. Bioinformatics.

[162] Cámara P.G., Levine A.J., Rabadán R. (2016). Inference of ancestral recombination graphs through topological data analysis.. PLOS Comput. Biol..

[163] Shull G.H. (1914). Duplicate genes for capsule-form inBursa bursa-pastoris.. Mol. Genet. Genomics.

[164] Davenport C.B. (1908). Degeneration, albinism and inbreeding.. Science.

[165] East E.M. (1908). Report of The Connecticut Agricultural Experiment Station.

[166] Shull G.H (1908). The composition of a field of maize.. J. Heredity.

